# Solvent-Driven Self-Organization of *Meso*-Substituted Porphyrins: Morphological Analysis from Fluorescence Lifetime Imaging Microscopy

**DOI:** 10.1021/acs.langmuir.2c03468

**Published:** 2023-04-12

**Authors:** Telma Costa, Mariana Peixoto, Marta Pineiro, J. Sérgio Seixas de Melo

**Affiliations:** CQC-IMS, Department of Chemistry, University of Coimbra, Coimbra P-3004-535, Portugal

## Abstract

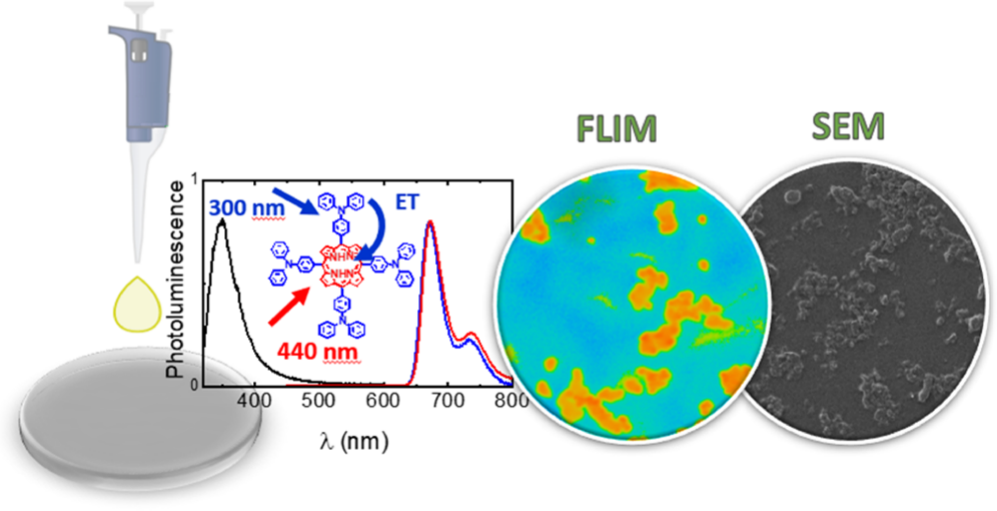

A morphological analysis of different thin films of *meso*-tetra-*p*-(di-*p*-phenylamino)phenylporphyrin, **H**_**2**_**T(TPA)**_**4**_**P**, was made by fluorescence lifetime imaging microscopy (FLIM) and scanning electron microscopy (SEM). A comprehensive study of **H**_**2**_**T(TPA)**_**4**_**P** was undertaken through UV/vis absorption and fluorescence techniques in different solvents, solvent mixtures and in thin films. In solution, occurrence of intramolecular energy transfer from the triphenylamine (TPA) moieties to the porphyrin core, with quenching efficiencies in the order of 94–97%, is observed. The energy transfer rate constants are determined assuming Förster’s dipole–dipole and Dexter’s electron exchange mechanisms. In drop-cast-prepared thin films, from samples with different solvent mixtures, the photoluminescence (PL) quantum yield (Φ_PL_) decreases ∼1 order of magnitude compared to the solution behavior. FLIM and SEM experiments showed the self-organization and morphology of **H**_**2**_**T(TPA)**_**4**_**P** in thin films to be highly dependent on the solvent mixture used to prepare the film. In chloroform, the solvent’s evaporation results in the formation of elongated and overlapped microrod structures. Introduction of a cosolvent, namely, a polar cosolvent, promotes changes in the morphology of the self-assembled structures, with the formation of three-dimensional spherical structures and hollow spheres. **H**_**2**_**T(TPA)**_**4**_**P** dispersed in a polymer matrix shows enhanced Φ_PL_ values when compared to the drop-cast films. FLIM images showed coexistence of three different states or domains: aggregated, interface, and nonaggregated or less-aggregated states. This work highlights the importance of FLIM in the morphological characterization of heterogeneous films, together with the photophysical characterization of nano- and microdomains.

## Introduction

Porphyrins constitute a class of molecules with widespread applications, ranging from medicine^1−3^ to material sciences with applications in organic photovoltaics,^4,5^ molecular electronics,^6^ and catalysis.^7−11^ The available functionalization sites (*meso*- and β-positions) and capability of coordinating with most of the periodic-table metals allows fine tuning of their optical, electrochemical, and physical properties (e.g., solubility, melting point), making them appealing candidates for a broad range of applications. Functionalization of the porphyrin core also allows modulation of the self-assembly processes. Porphyrins tend to self-assemble through weak intermolecular interactions such as π–π stacking, hydrogen bonding, metal coordination, hydrophobic effect, and electrostatic forces, leading to different types of excitonic coupling, namely, J-aggregates (side-to-side coupling of transition dipoles) and H-aggregates (face-to-face coupling) with red and blue shifts of the Soret and Q absorption bands, respectively. J-aggregates are known to allow faster energy transport due to the strong intermolecular coupling. This shows that modulation of the self-assembly processes, in order to control packing and film morphology, is of high relevance for applications involving solid or aggregate states. This includes optolectronic devices, since the general performance of these is highly influenced by the molecular packing that can be significantly affected by the intermolecular interactions established in the solid state. Nagarajan et al.^12^ reported the effect of the thin-film surface morphology on the performance of organic field-effect transistor (OFET) devices. The highest charge carrier mobility (4.4 cm^2^/V s) and a high on/off ratio (10^7^) were attained for *meso*-triarylamine porphyrin that showed the strongest intermolecular interactions. Triarylamine chromophores show a strong electron-donating ability and have been frequently used to modulate and enhance the optoelectronic properties of porphyrins and metalloporphyrins.^13−15^ The triphenylamine (TPA) substituent at the *meso*-position of the porphyrin ring acts as an antenna, and energy transfer from the TPA moiety to the porphyrin ring leads to an enhancement of the fluorescence quantum efficiency that increases with the number of TPA substituents.^16^

**Figure 1:**
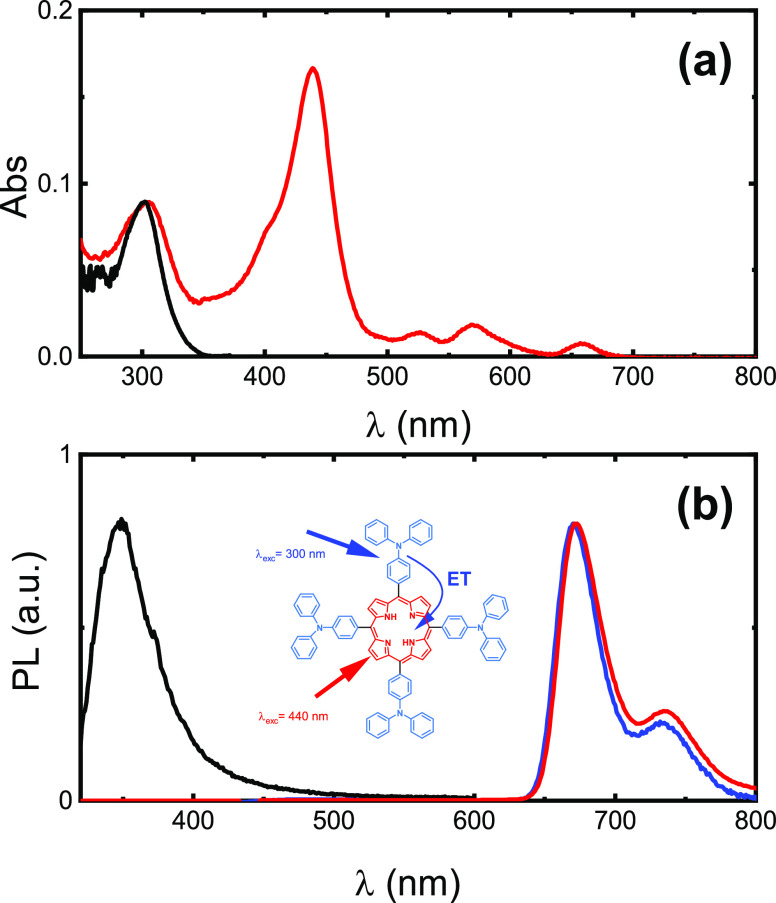
(a) Absorption and (b) PL spectra of **H**_**2**_**T(TPA)**_**4**_**P** obtained with λ_exc_ = 440 nm (red line) and λ_exc_ = 300 nm (blue line) in toluene. The normalized absorption and emission (λ_exc_= 300 nm) of TPA in toluene are also presented (black line). The inset figure schematically illustrates the energy transfer (ET) from TPA to the porphyrin core unit.

**Scheme 1:**
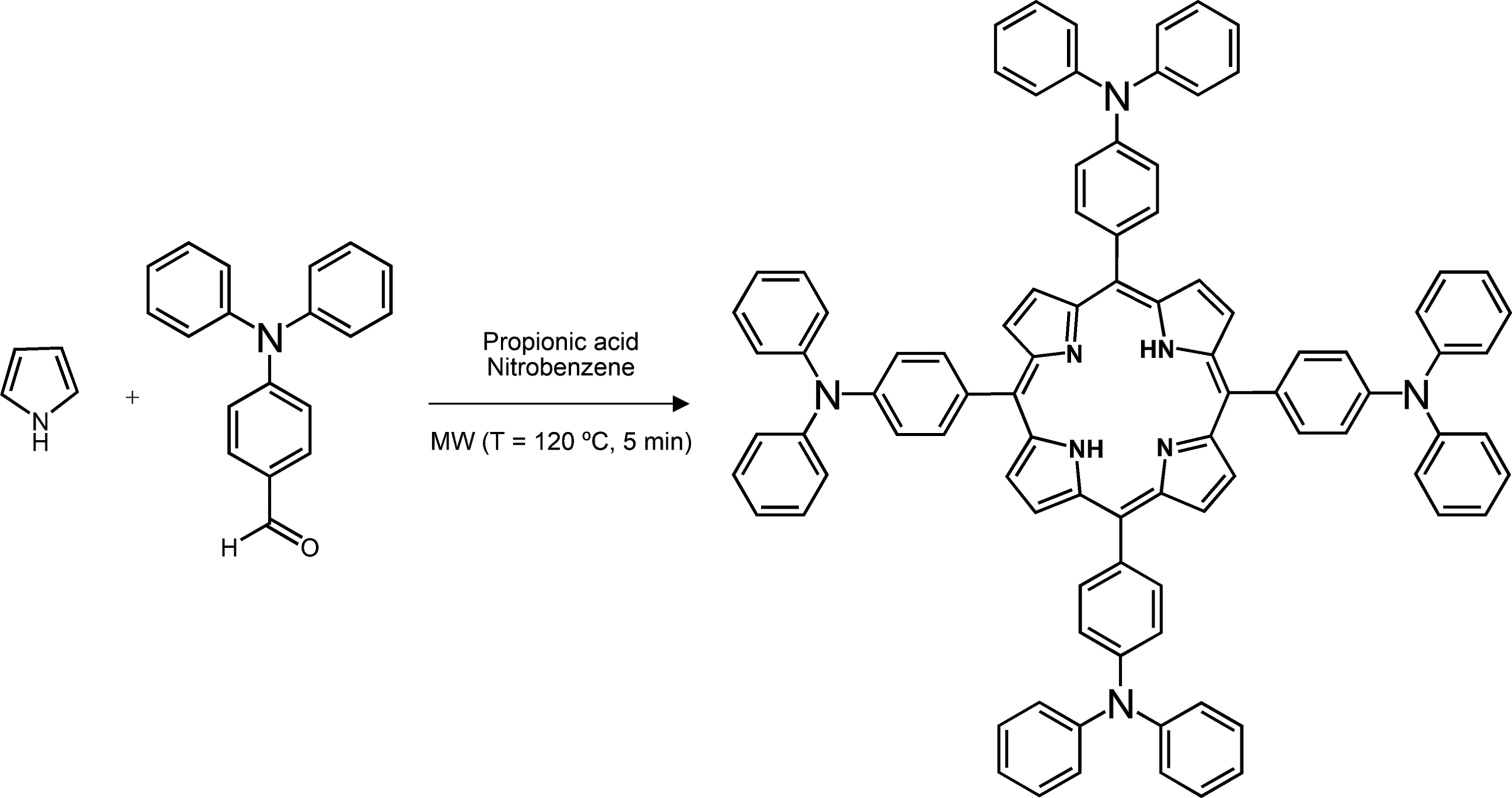
Synthesis of *meso*-tetra*-p*-(di*-p-*phenylamino)phenylporphyrin, **H_2_T(TPA)_4_P**

The morphology of the self-assembled structures depends, not only on its molecular structure but also on the methodology and solvent used for film deposition. The self-assembly of the porphyrins can be induced by the presence of a “bad–good” solvent and has shown to lead to the formation of different molecular structures with defined morphology, which can be controlled through the composition of the solvent mixture.^17^ For instance, *meso*-(pentafluorophenyl)porphyrin^18^ showed a transition from well-defined microrods to octahedral crystals when the water fraction increases from 70 to 80%. Up to 90%, these assembled structures tend to aggregate, forming a network of fused particles. Atomic force microscopy (AFM), scanning electron microscopy (SEM), and transmission electronic microscopy (TEM) are well-established techniques in the morphological characterization of the self-assembled aggregates on solid surfaces.^19−21^ There are some examples of the advantages of FLIM in the study of biological systems,^22^ namely, in the study of aggregation/disaggregation of amyloid proteins.^23^ Nevertheless, the use of this technique is still rare in the field of materials science.^24^ In this work, we aim to use FLIM to examine solvent-driven self-assembled structures of porphyrin bearing triphenylamine substituents, *meso*-tetra*-p*-(di*-p-*phenylamino)phenylporphyrin, **H**_**2**_**T(TPA)**_**4**_**P** (inset of Figure 1b and Scheme 1), and the characterization of the film morphology.

## Results and Discussion

### Photophysical Characterization in Solution

UV–visible absorption and photoluminescence (PL) spectra of **H**_**2**_**T(TPA)**_**4**_**P** porphyrin in toluene are shown in Figure 1, and the photophysical properties summarized in Table 1 (see spectra in different solvents in Figure S1). The absorption spectra of **H**_**2**_**T(TPA)**_**4**_**P** are characterized by the nonresolved absorption band at ∼300 nm and the typical absorption features of the porphyrin chromophore: Soret and Q_x,y,z_ bands. The band at 300 nm is absent in the absorption spectra of the porphyrin without substituent groups^25^ or in *meso*-tetraphenylporphyrin,^26^ indicating that this absorption band is due to the TPA groups introduced in the *meso*-position of the porphyrin ring. This assignment is further supported by the fact that this band matches the experimental and predicted absorption spectra of TPA as can be observed in Figures 1a and S2. The strong absorption at ∼436 to 441 nm is associated to the Soret or B band (S_2_ ← S_0_ transition), and the three Q bands in the visible region of the spectrum are attributed to the S_1_ ← S_0_ transitions.^26^ The PL spectra, obtained with the selective excitation of the porphyrin core (λ_exc_ = 440 nm), is characterized by the Q_x_*(0,0) emission band with a maximum at 667–683 nm and the red-shifted Q_x_*(0,1) band at 730–737 nm.^27,28^ The PL spectrum of TPA, a nonresolved emission band with maximum emission wavelength at 380 nm, is also shown in Figure 1b.^29^ The PL quantum yields (Φ_PL_) and the fluorescence decay times (τ) are presented in Table 1. In solution, the absolute Φ_PL_ values were found to be in the range of 0.17 (in MCH) to 0.22 (in THF), and the fluorescence decays, obtained with excitation at 460 nm (close to the Soret band and where the TPA unit does not absorb, see Figure 1), were found to be monoexponential in all solvents studied.

**Table 1:** Electronic Spectral (λ_Soret_, Wavelength Maximum of the Soret Band; λ_PL_^*Q*_x*_(0,0)^ and λ_PL_^*Q*_x*_(0,1)^ PL Wavelengths) and Photophysical Parameters (PL Quantum Yield, Φ_PL_, and Fluorescence Lifetime, τ_F_) for H_2_T(TPA)_4_P Obtained in Different Solvents^a^^b^

solvent^c^	λ_Soret_ (nm)	λ_PL_^Q_x*_(0,0)^ (nm)	λ_PL_^Q_x*_(0,1)^ (nm)	Φ_PL_ (λ_exc_ = 440 nm)	τ_F_ (ns)
CH_2_Cl_2_	438	677	737	0.210^b^	7.3
MCH	436	667	730	0.175^c^	7.8
CyHx	440	673	733	0.185^b^	10.5
THF	437	675	735	0.223^b^	9.7
DMF	437	678	730	0.185^c^	10.7
Dx	437	674	734	0.218^c^	9.4
CHCl_3_	439	676	736	0.213^b^	7.3
toluene	440	672	735	0.220^b^	9.7
DMSO	441	683	737	0.220^c^	7.3
powder				0.003^b^	

a MCH = methylcyclohexane; CyHx = cyclohexane; THF = tetrahydrofurane; DMF = dimethylformamide; Dx = dioxane; and DMSO = dimethylsulfoxide.

b Data obtained with the integrating sphere. See Experimental Methods for further details.

c Data obtained with ZnTPP (Φ_PL_= 0.030 in toluene)^30^ as a reference.

### Energy Transfer Mechanisms in Solution

The PL spectra obtained with λ_exc_= 300 nm, i.e., at the TPA absorption band, results in the appearance of a well-defined porphyrin emission band (TPA emission band is absent or with residual emission). Furthermore, when the excitation spectrum is collected at the porphyrin emission maximum, in addition to the characteristic band of porphyrin, the spectra show the characteristics of TPA absorption (Figure S2). These indicate efficient energy transfer from TPA to the porphyrin core in solution (inset of Figure 1b). To further support this hypothesis, the quenching efficiency (*Q*) and energy transfer rate constant (*k*_ET_) values, eqs 1 and 2, respectively, were determined in three different solvents^29^
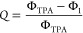
1and
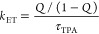
2where Φ_TPA_ and Φ_1_ stand, respectively, for the fluorescence quantum yields of TPA (donor) in the absence of an acceptor and of TPA in **H**_**2**_**T(TPA)**_**4**_**P** porphyrin; τ_TPA_ is for the singlet-state lifetime of the donor in the absence of an acceptor, and τ_1_ for the decay time of TPA that partially transfers energy to the porphyrin core, thus reducing its decay time component. The obtained results are presented in Table 2. The *Q* values were found to be solvent dependent, and in the order of 94–97%. Overall, the experimentally determined rate constant (eq 2), *k*_ET_, ranges from 1.49 × 10^10^ to 2.65 × 10^10^ s^–1^ (Table 2). The values are in the same order of magnitude of those previously found with triphenylamine-corrole dyads.^29^ Indeed, upon excitation, energy transfer may occur through Förster (dipole–dipole) or Dexter (electron exchange) mechanisms.^31^ According to the Förster mechanism, the energy transfer rate constant and energy transfer efficiency were calculated through eqs S1–S5 and the obtained results are presented in Table S1. In THF, the rate constant according to the Förster mechanism (eq S5), *k*_obs_^F^, is equal to 3.50 × 10^9^ s^–1^, which is considerably lower than the value experimentally observed in Table 2, *k*_ET_ = 1.49 × 10^10^ s^–1^. Moreover, the observed quenching efficiency from Föster mechanism, *Q*_obs_^F^ (=88.5%, eq S5 and Table S1) is lower than *Q* (=97%, eq 1 and Table 2). The observed differences suggest that other mechanism(s) may also be responsible for the fluorescence quenching in **H**_**2**_**T(TPA)**_**4**_**P**. The Dexter energy transfer mechanism involves a double electron exchange of the electron from the LUMO of the excited donor (TPA) to the empty LUMO of the acceptor (porphyrin ring) with a concomitant transfer of an electron from the HOMO of the acceptor to the HOMO of the donor. This mechanism requires short distances between the orbitals of the two moieties (donor and acceptor).

**Table 2:** Singlet-State Lifetimes of TPA in Solution (τ_TPA_) and in the H_2_T(TPA)_4_P Porphyrin (τ_1_), Quenching Efficiency *Q*, and Energy Transfer Rate Constant (*k*_ET_)

solvent^a^	Φ_1_	Φ_TPA_	*Q* (%)	τ_1_ (ns)	τ_TPA_ (ns)	*κ*_EΤ_ (10^10^ s^–1^)
THF (*n* = 1.4073, ε = 7.58)	0.0022	0.074	97	0.09	2.09	1.49
CH_2_Cl_2_ (*n* = 1.452, ε = 8.93)	0.0016	0.031	94	0.11	1.00	1.84
CyHx (*n* = 1.426, ε = 2.04)	0.0017	0.096	97	1.77	2.19	2.65

a *n* = refractive index and ε = solvent dielectric constant at 20 °C.

From TD-DTF calculations, the HOMO orbital is essentially located on the porphyrin moiety and on the four N-phenyl groups at the *meso*-position, whereas the HOMO – 4 on the core of the porphyrin. The LUMO and LUMO + 1 are mainly located on the porphyrin core (see Figures S3 and S4). These molecular orbitals are the main ones responsible for the electronic transitions in **H**_**2**_**T(TPA)**_**4**_**P**. Indeed, according to DFT calculations, in the porphyrin, these transitions involve the contribution from HOMO and HOMO – 4, and LUMO and LUMO + 1. The higher energy and oscillator strength transitions give rise to the Soret band, while the excited state with lower energy and smaller oscillator strength gives rise to the Q bands. Moreover, the predicted absorption spectrum of **H**_**2**_**T(TPA)**_**4**_**P** is found in good agreement with the experimental spectrum (in cyclohexane). According to the theoretical spectrum, there are three absorption bands: one located at the TPA core (around 310 nm) and two located at the porphyrin core. The first of these two bands is the Soret band, a strong and intense absorption peak that appears in the 424−428 nm range (*f* = 2.197–2.272). The second are Q bands, which are weaker and less intense than the Soret band and appear in the 604−666 nm range (*f* = 0.376–0.248).

The above data support that the LC-BPBE functional selected for the TD-DTF calculations correctly predicts the ground- and excited-state properties of **H**_**2**_**T(TPA)**_**4**_**P** in the studied solvents. This allows to correctly estimate the edge-to-edge distance between triphenylamine and the porphyrin, found to be 1.498 Å (Figure S3), suggesting that the Dexter mechanism can be present with **H**_**2**_**T(TPA)**_**4**_**P**. Furthermore, the Dexter energy transfer rate constant (*k*_ET_^D^, eq S6) can be calculated from the parameters given by eqs S7 and S8. Moreover, the contribution of the Dexter mechanism can also be estimated by the difference between *k*_ET_ and *k*_obs_^F^ (Table S1).^32,33^ The obtained Dexter rate constant (*k*_obs_^D^ = *k*_ET_ – *k*_obs_^F^) is found equal to 1.14 × 10^10^ s^–1^ in THF. The parameters (Φ_1_, Φ_TPA_, τ_1_, τ_TPA_, *R*_0_) and the data obtained (*k*_obs_^F^, *k*_cal_^F^, *k*_ET_, and *k*_obs_^D^) in THF and other solvents can be found in Table S1.

### **H_2_T(TPA)_4_P** in Mixtures of Good/Bad Solvents

**Figure 2:**
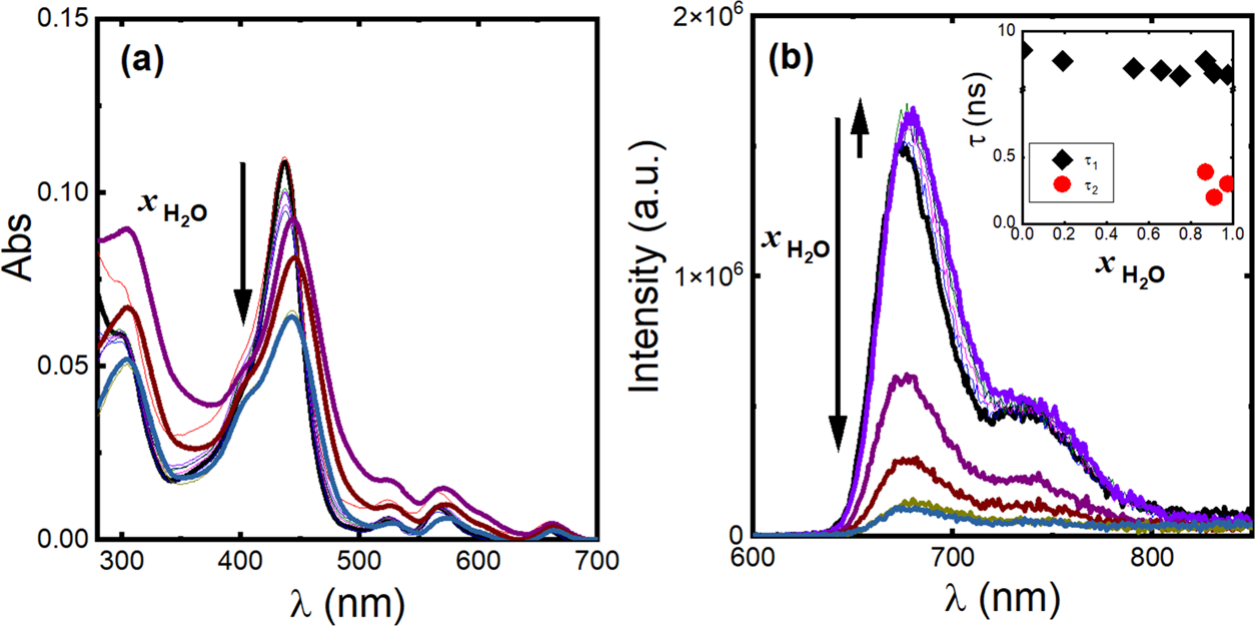
(a) Absorption and (b) PL (λ_exc_= 451 nm) spectra of **H**_**2**_**T(TPA)**_**4**_**P** in THF and THF/water mixtures. The dependence of the fluorescence decay times (τ_1_ and τ_2_) as a function of the molar fraction of water (*x*_H_2_O_) in THF/water solvent mixtures is shown as an inset in panel (b).

**H**_**2**_**T(TPA)**_**4**_**P** was further studied in good/bad (THF/water) solvent mixtures, with water inducing hydrophobic intermolecular interactions between **H**_**2**_**T(TPA)**_**4**_**P** molecules. The absorption bands at 437.5 nm (Soret), 566 nm [Q_x_(1–0)], and 641 nm [Q_y_(1–0)] in THF are red-shifted by 6, 8, and 17 nm, respectively, with increasing water molar fraction, *x*_H_2_O_ (Figure 2a); the bands at 524 nm [Q_x_(0–0)] and ∼600 nm [Q_y_(0–0)] show no significant shift (ca. 2 nm). A sharp decrease of the Soret absorption band value is also observed. Figure 2b shows the PL spectra of **H**_**2**_**T(TPA)**_**4**_**P** in different THF:water mixtures. At *x*_H_2_O_ < 0.8, there is a slight increase in the PL intensity with the fluorescence decays fitting monoexponentially with a decay time τ = 9.2–9.6 ns; at *x*_H_2_O_ > 0.8, the PL intensity drops, the PL spectra show a 6 nm red shift and the fluorescence decays become biexponential (τ_1_ = 9.2–9.4 ns and τ_2_ = 0.3–0.4 ns). Changes in the absorption, fluorescence intensity, and fluorescence decay times are assigned to aggregation of **H**_**2**_**T(TPA)**_**4**_**P** molecules promoted by the addition of water. Porphyrin molecules can arrange themselves in a head-to-tail or face-to-face alignment leading to the formation of J- or H-aggregates, respectively. Both aggregates are expected to cause changes in the absorption bands; whereas formation of H-aggregates gives rise to blue shifts of the absorption bands, J-aggregation leads to red-shifted absorption bands.^34−36^ Additionally, the fluorescence decay profiles remain single-exponential when H-aggregates are formed, whilst they become biexponential with J-aggregates, due to the strong electronic coupling between the chromophores.^35^ Our results corroborate previous findings that assigned these interactions (and spectral change) with the formation of J-aggregates. The increase in the water content in the mixture promotes the occurrence of aggregated structures oriented in a head-to-tail manner through π–π interactions and stabilization by hydrogen bonds. These structures lead to a decrease of the fluorescence intensity and fluorescence decay times when *x*_H_2_O_ ≥ 0.8, i.e., **H**_**2**_**T(TPA)**_**4**_**P** shows aggregation-caused quenching (ACQ) properties.

### Drop-Cast Films

The study of **H**_**2**_**T(TPA)**_**4**_**P** porphyrin in solution was extended to thin films. The application of porphyrins as donor or acceptor^37^ materials in optoelectronic devices demands the knowledge of their photophysical and self-assembly behavior in thin films and further optimization of film fabrication methods. The film formation methodology impacts the molecular packing orientation and charge transport properties of the material.^38^ The properties of the solvent or solvent mixture composition are known to affect the structure of the aggregates and therefore of J-aggregation.^17,39^

The **H**_**2**_**T(TPA)**_**4**_**P** porphyrin was studied in other 1:1 (v/v) solvent mixtures: CHCl_3_/cyclohexane (CHCl_3_/CyHx), chloroform/methanol (CHCl_3_/MeOH), and CHCl_3_/acetonitrile (CHCl_3_/CH_3_CN). **H**_**2**_**T(TPA)**_**4**_**P** is highly soluble in CHCl_3_ and CyHx, but shows reduced solubility in MeOH and CH_3_CN. Absorption and PL spectra obtained in CHCl_3_ and in these three solvent mixtures are shown in Figures S5 and S6 and the photophysical parameters are depicted in Table 3. These solutions were used to prepare drop-cast thin films on a quartz saphira substrate: films 1–4 in Table 3. Films were prepared by adding 10 μL of a solution of **H**_**2**_**T(TPA)**_**4**_**P** (1 × 10^–4^ M) onto a saphira disk at room temperature. By using different solvent mixtures, we play with the solvophobicity and the rate of evaporation of the solvent (b.p. = 61.2, 80.7, 64.7, and 82 °C for CHCl_3_, CyHx, MeOH, and CH_3_CN, respectively).

**Table 3:** Photophysical Parameters of H_2_T(TPA)_4_P Obtained in CHCl_3_: Solvent (Cyclohexane, CyHx; Methanol, MeOH; and Acetonitrile, ACN) Mixtures and in Thin Films (Films 1–4) Prepared from These Same Solutions.

	solvent (v/v)	λ_abs_ (nm)	λ_PL_ (nm)	Φ_PL_^a^	τ_1_ (ns)
solution	CHCl_3_	439	678	0.213	7.3
	CHCl_3_/CyHx (1:1)	441	676	0.152	8.0
	CHCl_3_/MeOH (1:1)	437	679	0.200	7.3
	CHCl_3_/ACN (1:1)	436	678	0.210	7.9
film 1	CHCl_3_	454	695	0.025	
film 2	CHCl_3_/CyHx (1:1)	447	686	0.023	
film 3	CHCl_3_/MeOH (1:1)	483	686	0.025	
film 4	CHCl_3_/ACN (1:1)	499	690	0.020	
film 5	Zeonex	441	667	0.103	

a Data obtained with an integrating sphere; see Experimental Methods for further details.

**Figure 3:**
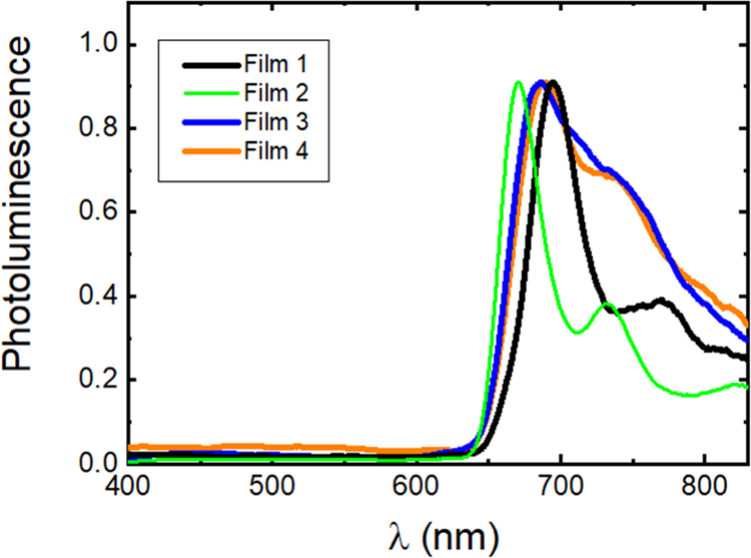
Normalized PL spectra (λ_exc_ = 440 nm) of **H_2_T(TPA)_4_P** films prepared by drop-cast of 1 × 10^–4^ M solution in CHCl_3_ (film 1), CHCl_3_/CyHx (film 2), CHCl_3_/MeOH (film 3), and CHCl_3_/CH_3_CN (film 4) solutions.

In thin films, the absorption and PL spectra are found strongly dependent on the solvent mixture used to prepare the film, which can anticipate its influence in the self-organization of **H**_**2**_**T(TPA)**_**4**_**P** in the solid state (Figures 3 and S7). In the absorption spectra of films 1–4, it is possible to identify the B and Q bands of **H**_**2**_**T(TPA)**_**4**_**P**, with these bands in films red-shifted relative to their absorption in solution (Table 3 and Figure S7).

The PL spectra of **H**_**2**_**T(TPA)**_**4**_**P** films collected at 300 and 440 nm (Figures S7 and 3, respectively) show the characteristic spectra of the porphyrin at 679–692 nm (dependent on the film morphology) and an additional less intense blue-shifted band in the wavelength range from 400 to 600 nm in film 3. The emission and excitation spectra of 4-(diphenylamino)benzaldehyde (TPA-CHO) thin film (see Figures S8 and S9) indicate that the low intensity band on the emission spectra of **H**_**2**_**T(TPA)**_**4**_**P** films, more intense in the film 3 spectrum, is most probably due to TPA emission. This has been previously observed with other triphenylamine-multibranching with porphyrins “core”.^40^ When the excitation spectrum is collected at the porphyrin emission maximum (λ_em_ = 680 nm), it matches the absorption spectra of **H**_**2**_**T(TPA)**_**4**_**P**, i.e., it shows the characteristic bands of the TPA absorption with porphyrin core, thus validating the occurrence of intramolecular energy transfer in thin films. The Φ_PL_ values significantly drop on going from solution to films, due to the ACQ behavior of the **H**_**2**_**T(TPA)**_**4**_**P** porphyrin (Table 3).

**Figure 4:**
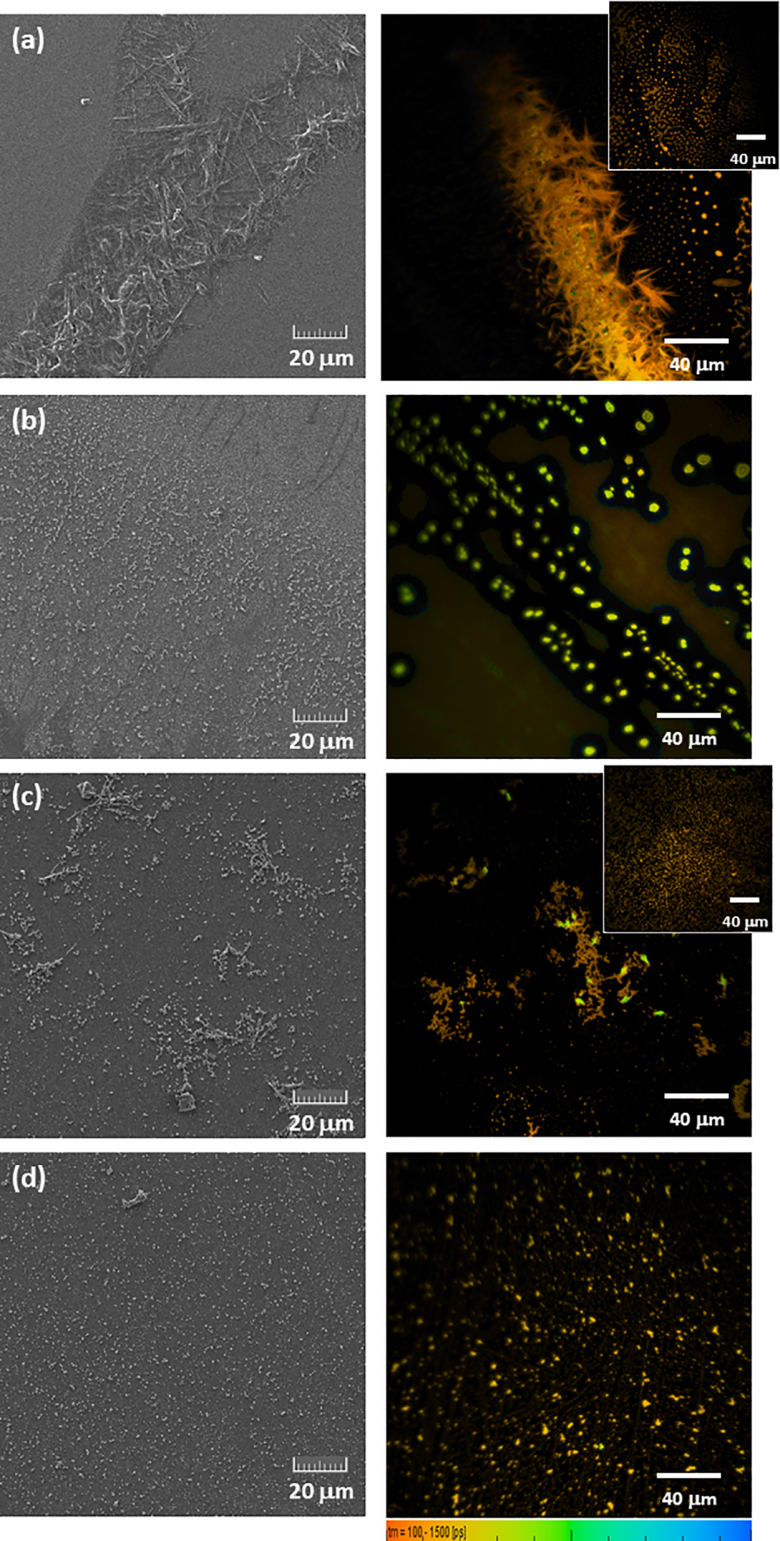
SEM (left-handed panels) and FLIM (right-handed panels) images of (a) film 1, (b) film 2, (c) film 3, and (d) film 4 (1 × 10^–4^ M of **H_2_T(TPA)_4_P** in solution). FLIM images of different regions of (a) film 1 and (c) film 3 are shown as insets. Obj. 40×/zoom 2.

To correlate the photophysical properties of the film with its morphology, the **H**_**2**_**T(TPA)**_**4**_**P** films were characterized by fluorescence lifetime imaging microscopy (FLIM) and scanning electron microscopy (SEM) (Figures 4 and S8–S13). SEM and FLIM images of film 1 are presented in Figure 4a. SEM shows formation of elongated and overlapped microrod structures, with mean widths of 0.26 and 0.46 μm (Figure S10). The thicker structures seem to result from the merge of two or more elongated structures. The FLIM image, obtained from the biexponential analysis of the fluorescence decays (Figure S11), provides a spatial/temporal view of the film. The image shows the presence of overlapped elongated structures coexisting with spherical aggregates (inset of FLIM image in Figure 4a). These smaller dimension aggregates were not detected in the SEM analysis, indicating that FLIM gives additional and complementary details of the film morphology.

Figure 4b–d shows the data of films obtained by the deposition of different 1:1 (v/v) solvent mixtures: CHCl_3_/CyHx (film 2), CHCl_3_/MeOH (film 3), and CHCl_3_/CH_3_CN (film 4). In film 2, both solvents are considered good solvents for **H**_**2**_**T(TPA)**_**4**_**P**, but the presence of CyHx induced formation of different nanostructures (Figure 4b). After evaporation of the solvent mixture, SEM images show formation of two-dimensionally (2D) spherical aggregates coexisting with fused spherical structures of different dimensions and shapes where it is possible to identify three average diameters: 0.16, 0.25, and 0.6 μm (Figure S12). Figure S12c shows the existence of elongated structures of fused particles with random sizes and shapes, which may result from the aggregation of spherical assembles or due to π–π stacking of adjacent self-assembled porphyrins during film formation.

In film 3, due to the boiling temperature of the solvent mixture, this becomes slightly affected when compared with the film obtained from CHCl_3_ alone; yet MeOH is not considered a good solvent for **H**_**2**_**T(TPA)**_**4**_**P**. SEM analysis of film 3 shows formation of well-defined spherical morphologies with diameters ranging from 0.200 to 0.600 μm (Figures 4c and S13), either dispersed in the film or in close proximity connected through an elongated structure. Formation of ring-like nanostructures is also observed (Figure S13c,d). The intermolecular hydrophobic π–π interactions between **H**_**2**_**T(TPA)**_**4**_**P** molecules lead to the formation of hollow spheres. In film 4 (Figure 4d), the spherical aggregates are dispersed on the quartz saphira substrate. It is possible to identify two average diameter distributions: 0.35 and 0.56 μm (Figure S14). It is also possible to find ring-like structures, similar to those found in film 3, but in much smaller amount. These results seem to indicate that formation of hollow spherical structures may be promoted by the presence of a polar solvent. Their formation was assigned to the nucleation process originated by 2D coordination interactions between adjacent porphyrins. These form 3D structures driven by the crystal lattice packing based on π–π stacking of 2D assembled layered structures during the self-assembly process.^41,42^ The spatial distribution of **H**_**2**_**T(TPA)**_**4**_**P** in films 2–4 is in good agreement with the morphological information retrieved from SEM images.

**Figure 5:**
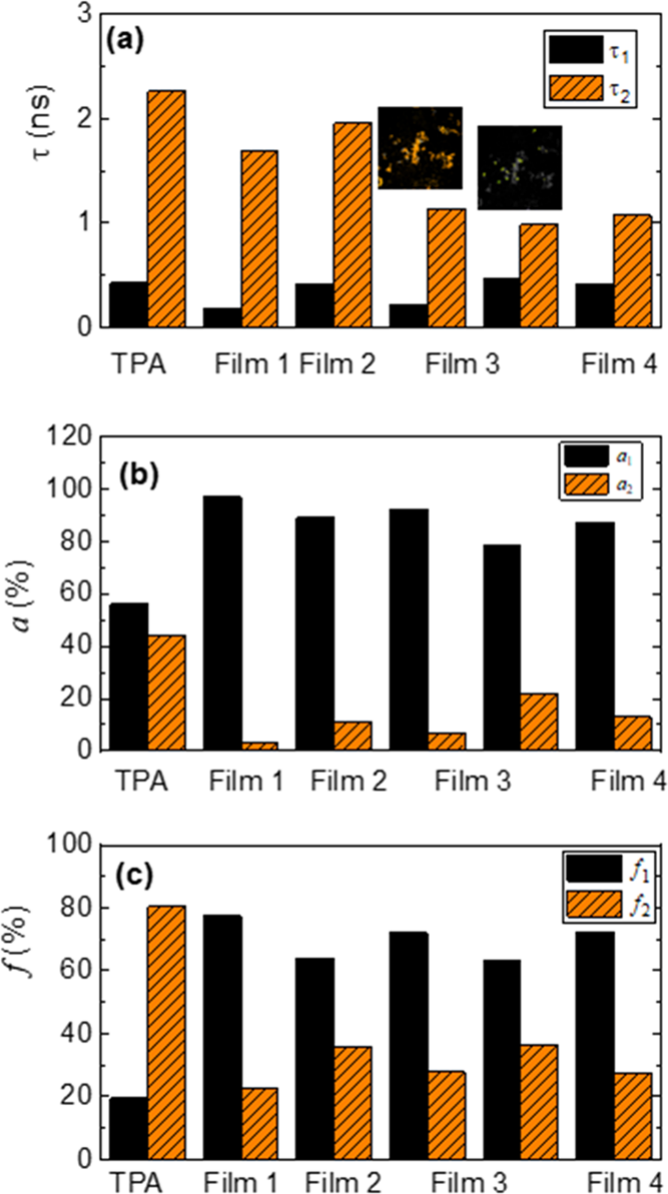
(a)Decay times (τ_1_ and τ_2_), (b) preexponential factors (*a*_1_ and *a*_2_), and (c) weighted preexponential factors of **H**_**2**_**T(TPA)**_**4**_**P** films. The data was retrieved from images presented in Figure 4. For film 3, the τ_i_, *a*_i_, and *f*_i_ values are presented for the two distinct regions shown in Figure 4c.

The time-resolved data (decay times, preexponential factors, and weighted preexponential factors) retrieved from the FLIM images presented in Figure 4 are shown in Figure 5. The fluorescence decays were fitted by a biexponential function with a shorter decay time (τ_1_) of ∼200 to 600 ps associated with a preexponential factor of ∼74 to 97%, and a longer decay time, ranging from ∼1000 to 2000 ps, with a lower preexponential factor (∼3 to 26%). For TPA films, the fluorescence decay times are 430 ps (56.7%) and 2.26 ns (43.6%), and for TPA-CHO films they are 648 ps (73%) and 3.40 ns (27%). These are significantly different from those obtained in films 1–4 meaning that these can be attributed to the porphyrin core, i.e., the shorter decay time may be assigned to the J-aggregates fluorescence^43−45^ and τ_2_ to the decay time of the porphyrin in film. The absence of the characteristic decay time of TPA indicates the occurrence of very efficient energy transfer from TPA to the **H**_**2**_**T(TPA)**_**4**_**P** aggregates.

**Figure 6:**
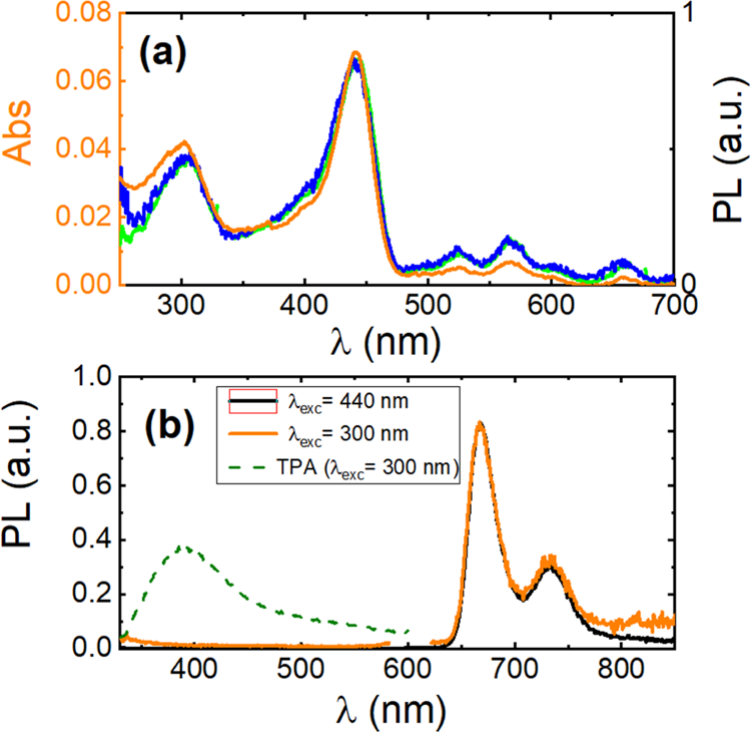
(a) Normalized absorption (orange line) and excitation (green line, λ_em_ = 667 nm and blue line, λ_em_ = 730 nm) spectra. (b) Normalized PL spectra of **H**_**2**_**T(TPA)**_**4**_**P** porphyrin (λ_exc_ = 300 and 440 nm) and **TPA** (λ_exc_= 300 nm) in Zeonex thin films.

**Figure 7:**
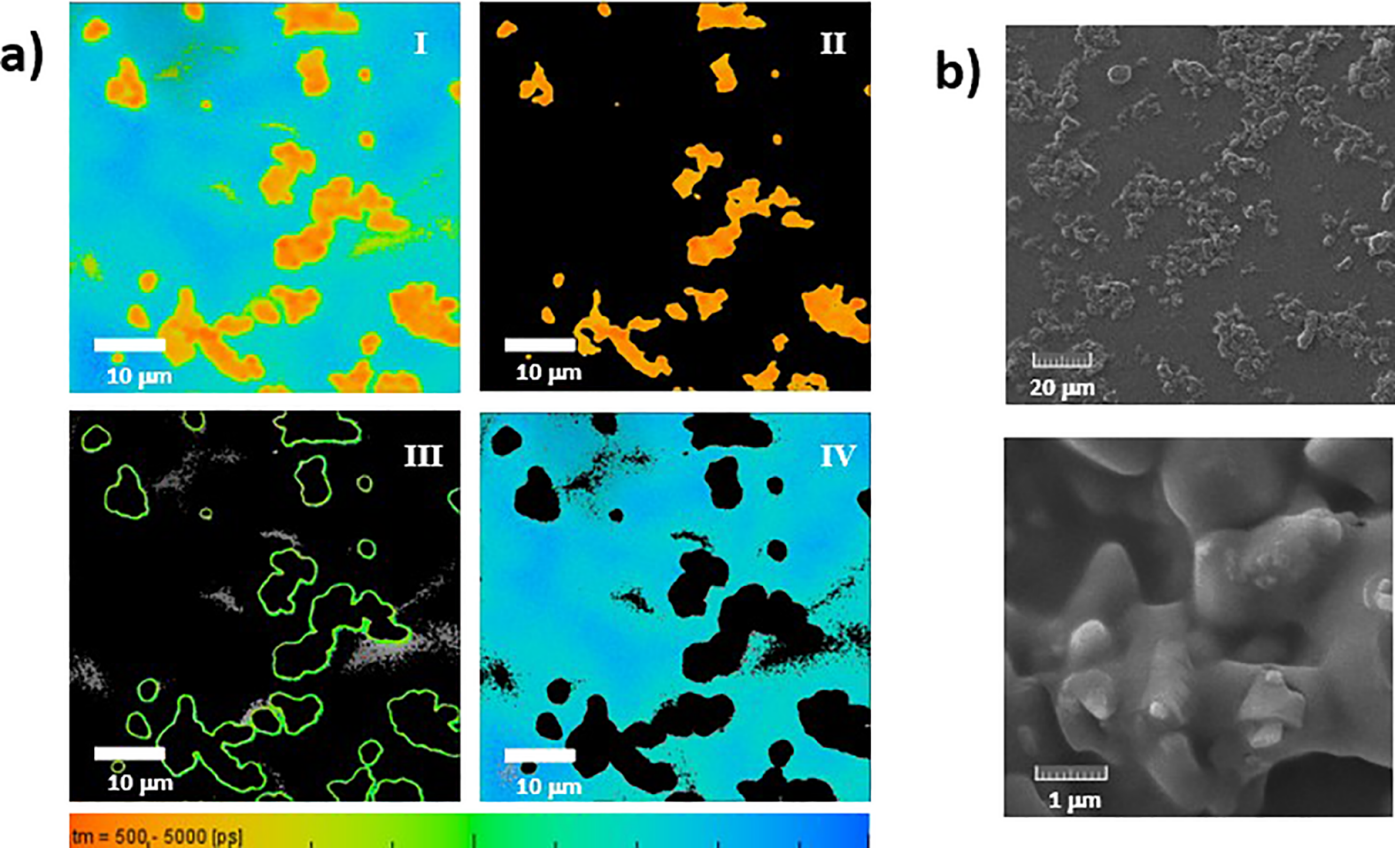
(a) FLIM image of **H**_**2**_**T(TPA)**_**4**_**P** in a Zeonex film (I) and deconvolution of FLIM image I (II–IV). Objective 40×/zoom 8; λ_exc_ = 375 nm. (b) SEM images of **H**_**2**_**T(TPA)**_**4**_**P** porphyrin dispersed in a Zeonex polymer matrix, obtained with different magnifications, 20 μm (top panel) and 1 μm (bottom panel).

**Figure 8:**
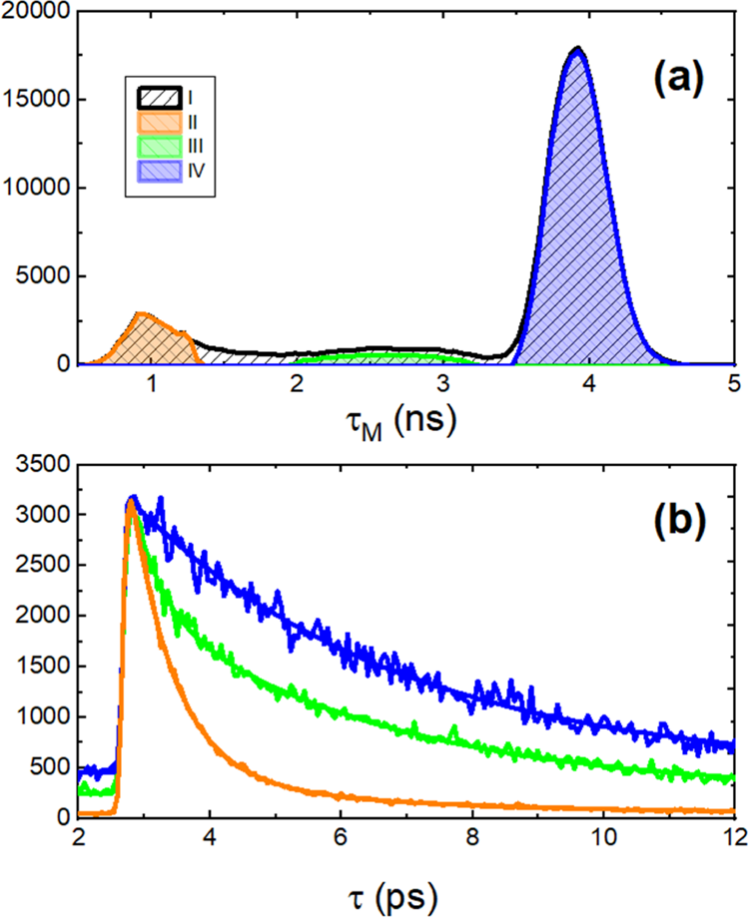
(a) Mean fluorescence lifetime (τ_M_) histogram of images I–IV of Figure 7. (b) Representative fittings and fluorescence decay profiles of images II–IV.

**Figure 9:**
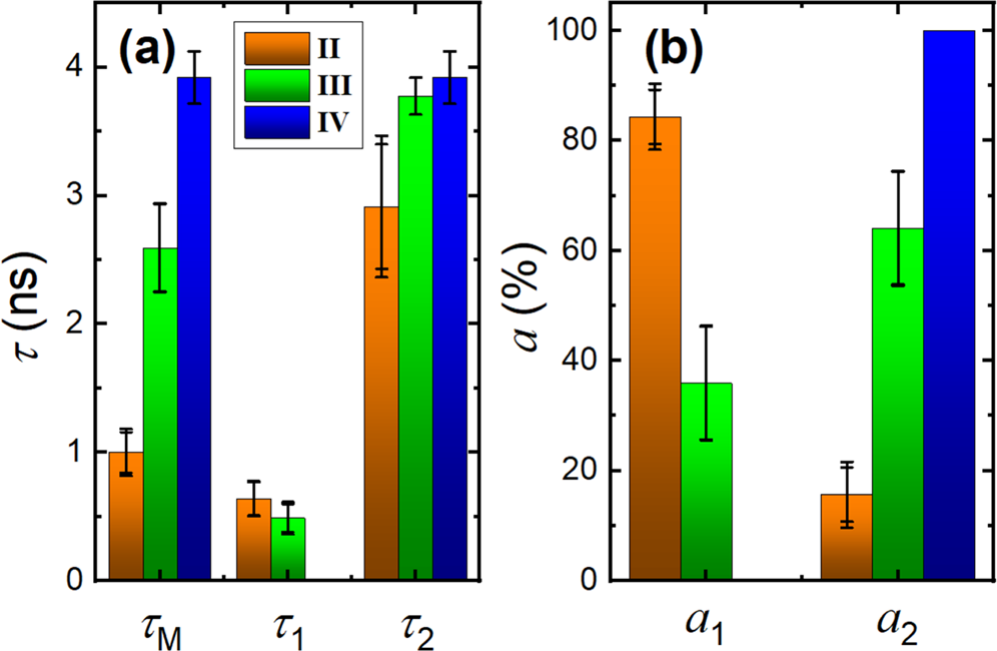
(a) Fluorescence lifetimes (τ_1_, τ_2_, and τ_M_) and (b) preexponential factors (*a*_1_ and *a*_2_) in the three different aggregation states presented in Figure 7(II–IV).

The presence of a cosolvent leads to an increase of the decay time τ_1_. However, the effect on τ_2_ seems to be dependent on the solvent; CyHx promotes an enhancement of τ_2_, whereas the presence of a bad solvent (MeOH or ACN) leads to its decrease. The preexponential factor *a*_1_ decreases in films 2–4 when compared to film 1, with the concomitant increase of *a*_2_. Even though τ_2_ decreases in films obtained from the deposition of good:bad solvent mixtures, the contribution of the longer decay time to the total decay, given by *f*_2_, increases (Figure 5c). Indeed, it occurs for the three solvent mixtures studied, regardless of the morphology of the film.

### Films in a Polymer Matrix

The absorption and PL (emission and excitation) spectra of **H**_**2**_**T(TPA)**_**4**_**P** in a polymer thin film (zeonex) are presented in Figure 6. The absorption spectra of **H**_**2**_**T(TPA)**_**4**_**P** dispersed in a polymer matrix are significantly different from the spectra obtained in the drop-cast films and similar to the spectra obtained in solution, evidencing a lower degree of **H**_**2**_**T(TPA)**_**4**_**P** aggregation. As a result, the PL quantum yield value is significantly enhanced when compared to the values obtained in drop-cast films (see Table 3). Nevertheless, J-aggregates are still present in Zeonex films, further enhancing the intensity at the red-edge of the excitation spectra.

The FLIM image of **H**_**2**_**T(TPA)**_**4**_**P** films (Figure 7, panel I) can be deconvoluted into three images associated with three different domains: aggregated, interface, and nonaggregated states (or less-aggregated state); in the lifetime color map are shown in orange, green, and blue, respectively. The three different states of aggregation have distinctive mean decay time (τ_M_) values and fluorescence decay profiles (Figure 8). The aggregates have different shapes and sizes (identified in Figure 7, panel II) and are associated to short-lived species (τ_M_ = 1.1 ns). At the interface, or external part of the aggregate (identified in Figure 7, panel III), the τ_M_ is found in the range of 2–3.6 ns. The longer lifetime (τ = 4.6 ns) is now found in the surroundings of the aggregates, which are probably associated with nonaggregated **H**_**2**_**T(TPA)**_**4**_**P** porphyrin dispersed in the polymer matrix or porphyrin in a lower degree of aggregation (identified in Figure 7, panel IV).

Figure 9 shows the mean decay times (τ_1_ and τ_2_) and preexponential factors obtained in the three different domains of aggregation shown in Figure 7(II–IV). The fluorescence decays of domains II and III are fitted with two exponentials: a fast decay time with a mean lifetime of 0.5–0.6 ns (τ_1_) and a longer lifetime, τ_2_. The shorter decay time is constant in domains II and III; nevertheless, *a*_1_ significantly decreases from 84.4 to 35%. On the other hand, the longer decay time, τ_2_, and its preexponential factor, *a*_2_, increase. In domain IV, the fluorescence decays can be nicely fitted with a monoexponential function and the obtained decay time is similar to the one found for τ_2_ in domain III. Indeed, τ_2_ increases on going from domain II to domain IV, and the concomitant increase of its contribution (*f*_2_) is observed: 45.8% (II) to 93.2% (III) to 100% (IV). Thus, τ_1_ and τ_2_ can be respectively assigned to the average decay time of porphyrin aggregates and isolated porphyrin molecules embedded into the polymer matrix.

Figures 7b and S15 show the SEM images of **H**_**2**_**T(TPA)**_**4**_**P** in zeonex films. The films show a different morphology compared to the one in the absence of a polymer matrix. The porphyrin seems to form amorphous aggregates embedded into the polymer matrix. Again, similar structures are observed from FLIM and SEM.

## Conclusions

From the present study, a new approach to the investigation of films (including of thin films) was pioneeringly introduced with the FLIM technique. In order to validate this approach, SEM studies were also conducted. A porphyrin-TPA (**H**_**2**_**T(TPA)**_**4**_**P**) structure, with solvent-dependent fluorescence properties, was used to characterize the morphological characteristics of the films, which were found dependent on the solvent mixture used (drop-cast preparation) or on the matrix (Zeonex polymer). In thin films prepared through drop-cast, the absorption and PL spectra show a red shift of absorption and emission bands. The magnitude of the shifts and the shape of the bands were found strongly dependent on the solvent mixture used to prepare the film, which resulted in the formation of different aggregated structures. Films prepared from polar solvents induced formation of hollow spherical structures, whereas from nonpolar solvent mixtures, the **H**_**2**_**T(TPA)**_**4**_**P** aggregates assemble into elongated structures with random sizes and shapes.

The control and tuning of the morphology of self-assembled nanostructures is very important to the development of new materials with application in materials science. We have shown that FLIM is a valuable technique in the study of these materials and provides new insights into the self-assembling of porphyrins on solid surfaces. FLIM shows many advantages in the study of organic and polymeric materials, especially in the case where different microdomains are present. The acquisition of a SEM image of an electrically nonconducting material is difficult and the most widely adopted strategy is to coat the material with a thin conducting film, such as an Au/Pd film, to obtain a good signal and minimize degradation caused by the electron beam. FLIM technique avoids any particular preparation step and coating, with the only condition that the probe used must be fluorescent. Sample damaging is then avoided and the characteristics of the sample are preserved.

## Experimental Methods

### Materials

All chemical were purchased from commercial suppliers (Sigma-Aldrich, Fischer Scientific, or TCI) and used without further purification. The solvents used in the photophysical studies were of spectroscopic grade or equivalent, and water was purified through a Millipore Milli-Q water purification system (18.2 MΩ·cm at 25 °C).

### Synthesis and Characterization

The *meso*-tetra*-p*-(di*-p-*phenylamino) phenylporphyrin, **H**_**2**_**T(TPA)**_**4**_**P**, was prepared based on a previous reported procedure.^46,47^ The reaction scheme is presented in Scheme 1. 4-(Diphenylamino)benzaldehyde (2.6 mmol) and pyrrole (2.6 mmol) were added to a mixture of propionic acid (0.93 mL) and nitrobenzene (0.4 mL) in a microwave tube. The reaction mixture was heated at 120 °C for 5 min under microwave irradiation (CEM apparatus). After cooling to room temperature, the black crude was purified by silica gel column chromatography using dichloromethane as the eluent. The red fraction was collected, evaporated under reduced pressure, and recrystallized in ethyl acetate/hexane, yielding the corresponding porphyrin as a crystalline dark purple solid (yield = 22%). The ^1^H NMR spectra of **H**_**2**_**T(TPA)**_**4**_**P** are shown in Figure S16 with the proton signals identified and are in agreement with the previously described study.^48^^1^H NMR (400 MHz, CDCl_3_) δ (ppm): 9.00 (s, 8H); 8.096–8.075 (d, 8H, *J* = 8.4 Hz); 7.473–7.412 (m, 32 H), 7.15 (m, 8H), −2.665 (s, 2H, NH).

### Absorption and Fluorescence

UV–vis absorption spectra measurements were acquired either on Cary 5000 UV–vis–NIR or Shimadzu UV-2450 double-beam spectrometers with a 1 cm quartz cuvette over the range of 200–800 nm. Fluorescence measurements were recorded with FluoroMax-4 or Horiba–Jobin–Ivon SPEX Fluorolog 3-22 spectrometers. Fluorescence spectra were corrected for the wavelength response of the system. Photoluminescence quantum yields (Φ_PL_), in solution and in the solid state, were measured using the absolute method with a Hamamatsu Quantaurus QY absolute photoluminescence quantum yield spectrometer, model C11347 (integrating sphere). For the Φ_PL_ measurements of solid-state thin films, a clean sapphire substrate was used as a reference.

### Nanosecond Time-Resolved Fluorescence

Fluorescence decays were measured using a home-built nanosecond time-correlated single photon counting (ns-TCSPC) apparatus described elsewhere.^49^ The excitation source used was a Horiba-JI-IBH NanoLED, λ_exc_ = 460 nm. The apparatus consists of a Jobin–Ivon H20 monochromator, a Philips XP2020Q photomultiplier, a Canberra instruments time-to-amplitude converter (2145), a Multichannel Analyser (AccuSpec), and START and STOP discriminators. The fluorescence decay curves were deconvoluted using the experimental instrument response function signal collected with a scattering solution (aqueous Ludox solution). The deconvolution procedure was performed using the modulating functions method of George Striker in the SAND program, and previously reported in the literature.^50^

### Fluorescence Lifetime Imaging Microscopy

Fluorescence lifetime images were collected by using a Becker and Hickl (GmbH) DCS-120 Confocal FLIM System. The system is equipped with a TCSPC System module (SPC-150N), a NIKON Ti2-U inverted optical microscope, controlled by a galvo-drive unit (Becker and Hickl GDA-121), and a hybrid GaAsP photodetector (300–720 nm) controlled by a DCC-100 detector controller card. Two objectives were used: 20× (CFI Plan-Achromat 20×/0.40/1.20) and 40× (CFI. Plan-Achromat 40×/0.65/0.56). The DCS-120 confocal microscope system is equipped with a polarizing beam splitter. The excitation source is a picosecond diode laser of 375 nm wavelength (bh BDL series lasers) working on a pulsed mode (repetition rate: 80 MHz). The IRF of the system is found to be less than 100 ps. The total laser power at the sample was set to 40% of the maximum value and the collected emission passed through a 1 nm pinhole and long pass filters 390LP and 520LP. The FLIM images were scanned and recorded at a resolution of 512 × 512 pixels using the “FIFO imaging” mode of the SPC-150N modules. Data analysis was performed through a SPCImage NG data analysis software. The decay curves were fitted using the maximum-likelihood algorithm (or maximum-likelihood estimation, MLE) fitting method in each pixel. The sapphire substrates were glued on a microscope slide and the measurements were performed by placing the inverted slide on the microscope stage.

### Scanning Electron Microscopy

The SEM/EDS analysis was carried out with a TESCAN Vega3 SBH SEM equipped with BSE (annular, YAG crystal, 0.1 atomic resolution), SE (Everhart–Thornley type, YAG crystal), current (pA meter), and EDS (Bruker Xflash 410 M) detectors, belonging to TAIL-UC. A 20 kV acceleration voltage was applied and data was obtained using a working distance of 15–15.1 mm. The magnification and pixel size varied from sample to sample. Before the measurements, the film samples were coated with a 10-nm-thick Au/Pd film.

### Time-Dependent Density Functional Theory (TDDFT) Calculations

All theoretical calculations were of the density functional theory (DFT) type and carried out using GAMESS-US version R3.^51^ A range-corrected LC-BPBE (ω = 0.20 au^–1^) functional, as implemented in GAMESS-US,^51^ was used in both ground- and excited-state calculations. TDDFT calculations, with similar functionals, were used to probe the excited-state potential energy surface (PES). A solvent was included using the polarizable continuum model with the solvation model density to add corrections for cavitation, dispersion, and the solvent structure. In TDDFT calculation of Franck–Condon excitations, the dielectric constant of the solvent was split into a “bulk” component and a fast component, which is essentially the square of the refractive index. Under “adiabatic” conditions, only the static dielectric constant was used. DFT and TDDFT calculations, for the location of critical points, was carried out using SBKJC ECPs (Stevens–Bash–Krauss–Jasien–Cundari Effective Core Potentials for nonvalence electrons with a split-31G for valence electrons).^52−54^ For the resulting optimized geometries, time-dependent DFT calculations (using the same functional and basis set as those in the previous calculations) were performed to predict the vertical electronic excitation energies. Frequency analyses for each compound were also computed and did not yield any imaginary frequencies, indicating that the structure of each molecule corresponds to at least a local minimum on the potential energy surface.

## Supporting Information Available

Absorption and PL spectra of H_2_T(TPA)_4_P (λ_exc_= 440 and 300 nm) in different solvents; calculated and experimental absorption spectra of TPA and excitation spectrum of H_2_T(TPA)_4_P collected at 675 nm; rate constants for Forster and Dexter mechanisms; singlet-state lifetimes of TPA in solution (τ_TPA_) and of TPA in the H_2_T(TPA)_4_P porphyrin (τ_1_), quenching efficiency *Q*, and energy transfer rate constants; optimized geometry of H_2_T(TPA)_4_P at the level theory of DFT//LC-BPBE (ω = 0.2)/SBKJC; HOMOs and LUMOs of H_2_T(TPA)_4_P; absorption spectra of H_2_T(TPA)_4_P in CHCl_3_ and in 1:1 (v/v) CHCl_3_/CyHx, CHCl_3_/MeOH, and CHCl_3_/CH_3_CN mixtures; normalized fluorescence emission spectra obtained at λ_exc_= 440 and 300 nm of films 1–4; normalized absorption and excitation spectra of H_2_T(TPA)_4_P films 1–4; emission spectra of a 4-(diphenylamino)benzaldehyde (TPA-CHO) film; normalized excitation spectra obtained at λ_em_= 450 nm film 3 and TPA-CHO thin films; SEM of films 1–5; single-pixel fluorescence decay of H_2_T(TPA)_4_P aggregate retrieved from FLIM data; and ^1^H NMR spectrum of H_2_T(TPA)_4_P in CDCl_3_ (PDF)
