# Supplementary material for: Solvent-Driven Self-Organization of Meso-Substituted Porphyrins: Morphological Analysis from Fluorescence Lifetime Imaging Microscopy
Source: Langmuir. 2023 Apr 12;39(16):5727–37. doi: 10.1021/acs.langmuir.2c03468 (PMC10134502; doi:10.1021/acs.langmuir.2c03468)
Supplement: Supplementary file 1 — la2c03468_si_001.pdf [file la2c03468_si_001.pdf]

# Supplementary Information

# Solvent-driven self-organization of meso-substituted porphyrins: morphological analysis from Fluorescence Lifetime Imaging Microscopy.

Telma Costa*, Mariana Peixoto, Marta Pineiro and J. Sérgio Seixas de Melo

*University of Coimbra, CQC-IMS, Department of Chemistry, Coimbra P-3004-535, Portugal.*

*KEYWORDS. FLIM, Porphyrin, Film morphology, energy transfer, SEM, Fluorescence.*

E-mail: tcosta@qui.uc.pt

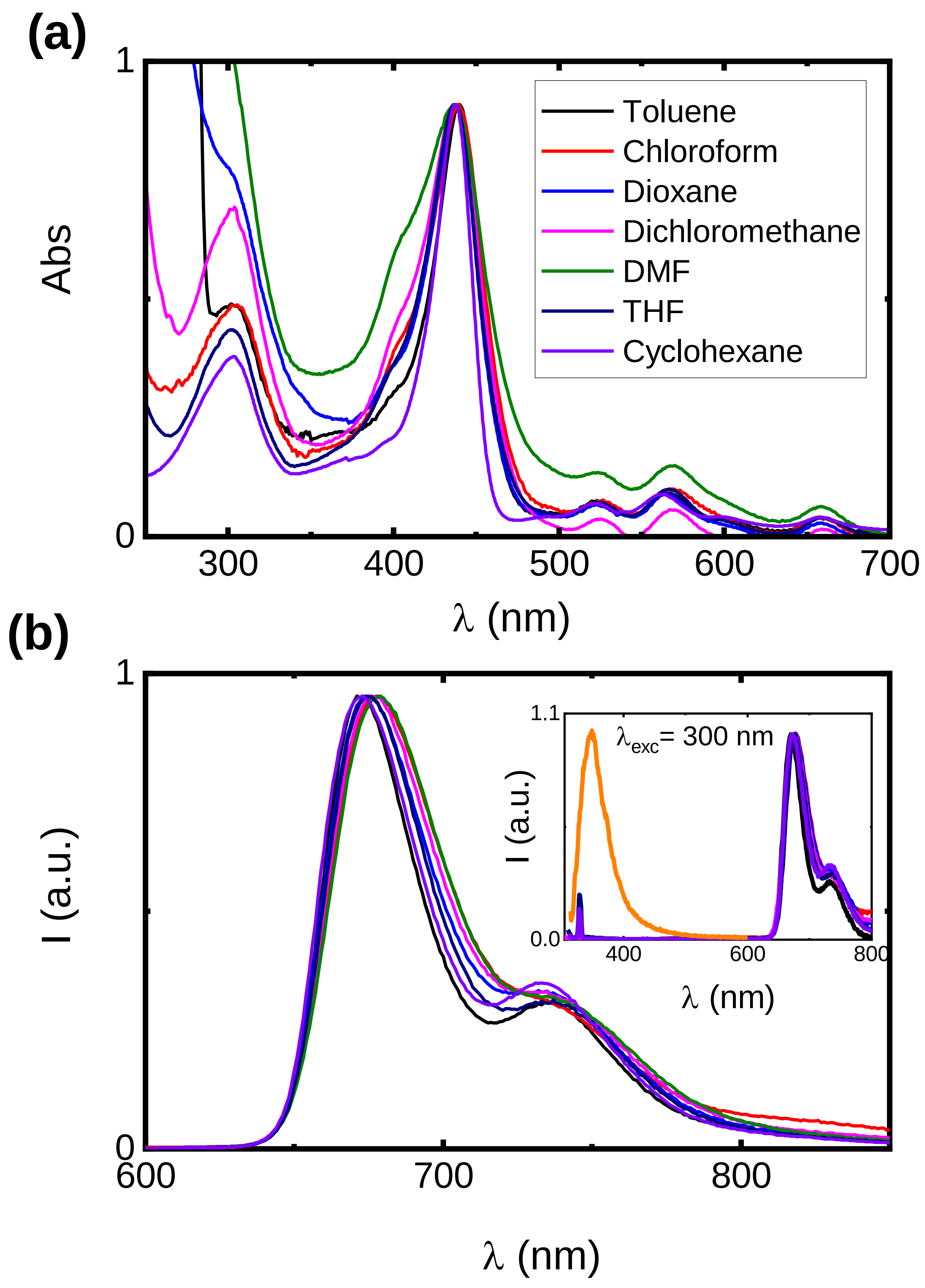


**Figure S1.** Absorption **(a)** and PL spectra **(b)** of $H_2T(TPA)_4P$ obtained with $\lambda_{exc}$= 440 nm and 300 nm (inset of (b)) in different solvents. The absorption and PL ($\lambda_{exc}$= 300 nm) spectra of TPA in toluene are also presented.

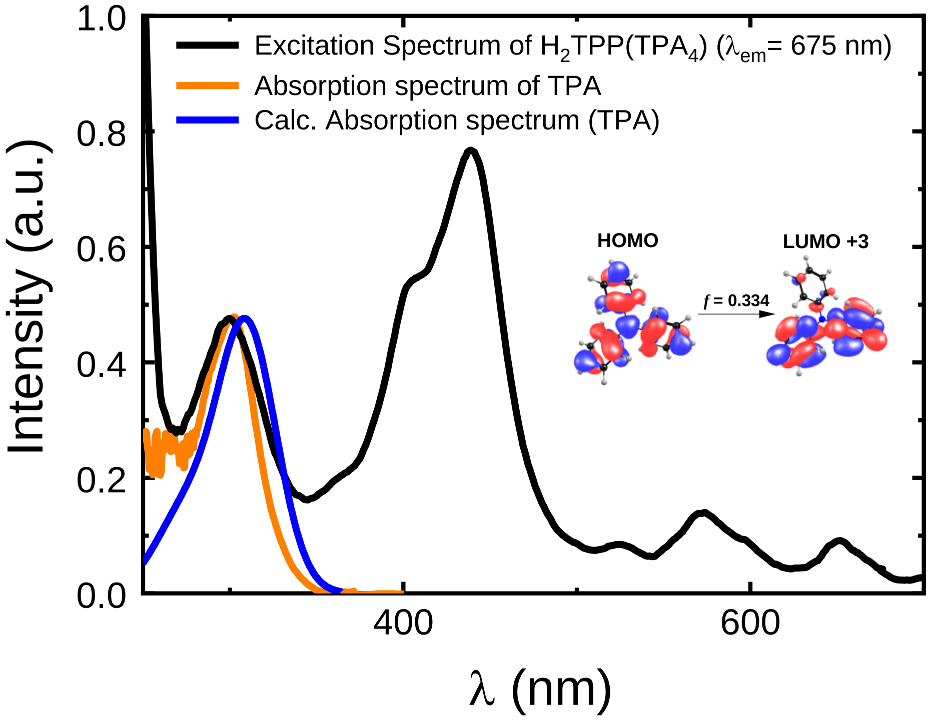


**Figure S2.** Calculated and experimental absorption spectrum of TPA and excitation spectrum of $\mathbf{H_2T(TPA)_4P}$ collected at 675 nm. Shown as inset are the HOMO and LUMO orbitals of **TPA**.

### Rate Constants for the Forster and Dexter mechanisms

The rate constant of energy transfer assuming a Förster mechanism is given by:[1]

$$k_{ET}^{Förster} = \frac{9000 \ln 10}{128\pi^5 N_A} \frac{k^2 \phi_D}{n^4 \tau_D R^6} \underbrace{\int_0^\infty F_D(\bar{\upsilon}) \varepsilon_A(\bar{\upsilon}) \lambda^4 \mathrm{d}(\bar{\upsilon})}_{J^F(\bar{\upsilon})} \qquad \text{(Eq. S1)}$$

where $N_A$ is the Avogadro's number, $\phi_D$ and $\tau_D$ are the fluorescence efficiency and radiative lifetime of the donor (D), i.e., of TPA, $k$ is an orientation factor relating the geometry of the D–A dipoles, $n$ the refractive index of the media, $J^F(\bar{\upsilon})$ the overlap integral, $F_D(\bar{\upsilon})$ the spectral distribution of donor emission and $\varepsilon_A(\bar{\upsilon})$ the molar extinction of the acceptor ($\mathbf{H_2T(TPA)_4P}$). The $\kappa^2$ values vary in a range of 0, if the electric field of the excited donor and acceptor's absorption dipole are perpendicular, to 4, if they are collinear. For random geometry, as obtained in solutions and/or gaseous systems, $k^2$ is 2/3. The rate constant for energy transfer can also be calculated in terms of the critical transfer distance parameter, $R_0$:[1]

$$k_{cal}^F = \frac{1}{\tau_D}\left(\frac{R_0}{R}\right)^6 \qquad \text{(Eq. S2)}$$

where

$$R_0(\dot{A}) = 0.211[k^2\, n^{-4}\, \phi_D\, J(\lambda)]^{\frac{1}{6}} \qquad \text{(Eq. S3)}$$

The critical energy transfer distance ($R_0$), in the three solvents studied, ranges from 21 to 31 Å (see Table S1), which is considerably larger than the center-to-center donor–acceptor distance ($R$ = 9.28 Å). The Förster energy transfer efficiency $\left(Q_{cal}^F\right)$ is similar to $Q$, but is consistently higher than $Q_{obs}^F$ (Eq. S5):

$$Q_{cal}^F = \frac{\left(\frac{R_0}{R}\right)^6}{1-\left(\frac{R_0}{R}\right)^6} \qquad \text{(Eq. S4)}$$

According to Förster mechanism, the observed energy transfer rate constant is given by:

$$k_{obs}^F = \frac{1}{\tau_D}\left(\frac{Q_{obs}}{1-Q_{obs}}\right) \qquad \text{(Eq. S5)}$$

where $Q_{obs}$ is calculated through the overlap between the normalized excitation and the absorption spectra. The obtained values suggest the occurrence of intramolecular energy transfer with efficiencies of 66%-88.5 % ($Q_{obs}^F$ in Table S1), which are significantly lower than $Q$. The $k_{ET}$ values are higher than both $k_{obs}^F$.

Dexter energy transfer mechanism involves a double electron exchange, where the electron from the LUMO of the excited donor to the empty LUMO of the acceptor with a concomitant transfer of an electron from the HOMO of the acceptor to the HOMO of the donor. This is a short distance mechanism, whose rate is expected to show an exponential dependence with the donor-acceptor distance.[1] The estimated edge-to-edge distance (R) between triphenylamine and the porphyrin is 1.497 Å. The short distance between the two moieties may facilitate donor-acceptor orbital interactions and promote electron exchange process. The rate of energy transfer by Dexter mechanism is determined using the following equation:[2]

$$k_{ET}^D = \frac{4\pi^4 H_{DA}^2}{h} J^D(\bar{\upsilon}) \qquad \text{(Eq. S6)}$$

where

$$J^D(\bar{\upsilon}) = \frac{\int_0^\infty F_D(\bar{\upsilon})\varepsilon_A(\bar{\upsilon})\mathrm{d}(\bar{\upsilon})}{\int_0^\infty F_D(\bar{\upsilon})\mathrm{d}(\bar{\upsilon}) \int_0^\infty \varepsilon_A(\bar{\upsilon})\mathrm{d}(\bar{\upsilon})} \qquad \text{(Eq. S7)}$$

$H_{DA}$ is the electronic coupling matrix and $J^D$ is the Dexter overlap integral. The electron coupling matrix can be calculated considering two states of formulation ($S_0$ and $S_n$, through the generalized Mulliken-Hush model:

$$H_{DA} = \frac{\mu_u \Delta E}{\sqrt{(\Delta\mu)^2 + 4(\mu_{\mathrm{tr}})^2}} \quad \text{(Eq. S8)}$$

$\mu_{\mathrm{tr}}$ is the transition dipole moment calculated for a particular excited state, $\Delta\mu$ is the transition dipole moment between the ground and the excited states and $\Delta E$ is the vertical excitation energy. Alternatively, the contribution of the Dexter mechanism can be determined by the difference between $k_{obs}$ and $k_{obs}^F$ (Table S1).[2,3]

**Table S1. Singlet-state lifetimes of TPA in solution ($\tau_{TPA}$) and of TPA in the $H_2T(TPA)_4P$ porphyrin ($\tau_1$), quenching efficiency $Q$ and energy transfer rate constant constants.**

| ***Solvent**** | $\Phi_1$ | $\Phi_{TPA}$ | $\tau_1$ **(ns)** | $\tau_{TPA}$ **(ns)** | $Q$ **(%)**[a] | $k_{ET}$ [b] **($10^{10}$ $s^{-1}$)** | $R_0$ **(Å)** [c] | $Q^F_{obs}$ (%) [d] | $k^F_{obs}$ **($10^{10}$ $s^{-1}$)** [e] | $Q^F_{cal}$ (%) [f] | $k^F_{cal}$ **($10^{10}$ $s^{-1}$)** [g] | $k_{obs} - k^F_{obs}$ **($10^{10}$ $s^{-1}$)** | $k_{diff}$ **($10^{10}$ $Lmol^{-1}s^{-1}$)** [h] |
|---|---|---|---|---|---|---|---|---|---|---|---|---|---|
| THF ($n$ = 1.4073, ε = 7.58) | 0.002 2 | 0.074 | 0.09 | 2.09 | 97 | 1.49 | 31.2 | 88.5 | 0.35 | 99.9 | 65.5 | 1.44 | 1.30 |
| $CH_2Cl_2$ ($n$ = 1.452, ε = 8.93) | 0.001 6 | 0.031 | 0.11 | 1.00 | 94 | 1.84 | 27.5 | 66.4 | 0.197 | 99.9 | 68.3 | 1.64 | 1.50 |
| CyHx ($n$ = 1.426, ε = 2.04) | 0.001 7 | 0.096 | 1.77 | 2.19 | 97 | 2.65 | 21.6 | 80.6 | 0.199 | 99.4 | 7.54 | 2.46 | 0.67 |

[a] Quenching efficiency calculated using Eq. 1 (main text).
[b] Observed energy transfer rate constant calculated using Eq. 2 (main text).
[c] Critical energy transfer distance calculated using Eq. S3.
[d] $Q^F_{obs}$ is calculated through the overlap between the normalized excitation and absorption spectra.
[e, g] Rate constants for energy transfer calculated using Eqs. S5 and S2, respectively.
[f] Calculated using Eq. S4.
[f] Diffusion controlled rate constants (T= 20 ºC) from ref. [4].

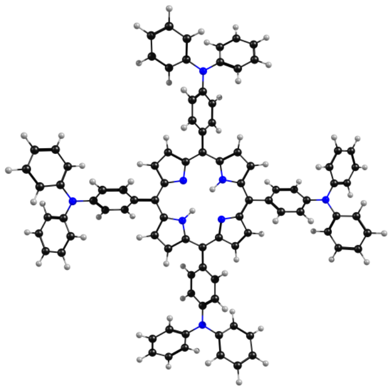

**Figure S3.** Optimized geometry of $\mathbf{H_2T(TPA)_4P}$ at the level theory of the DFT//LC-BPBE($\omega$=0.2)/SBKJC.

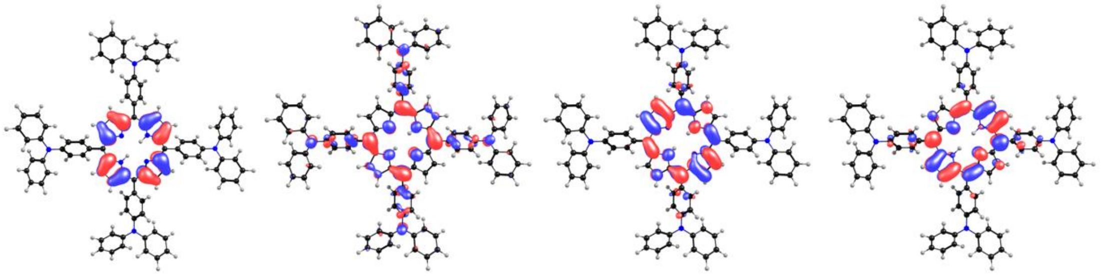

**HOMO-4** **HOMO** **LUMO** **LUMO +1**

**Figure S4.** HOMOs and LUMOs of $\mathbf{H_2T(TPA)_4P}$.

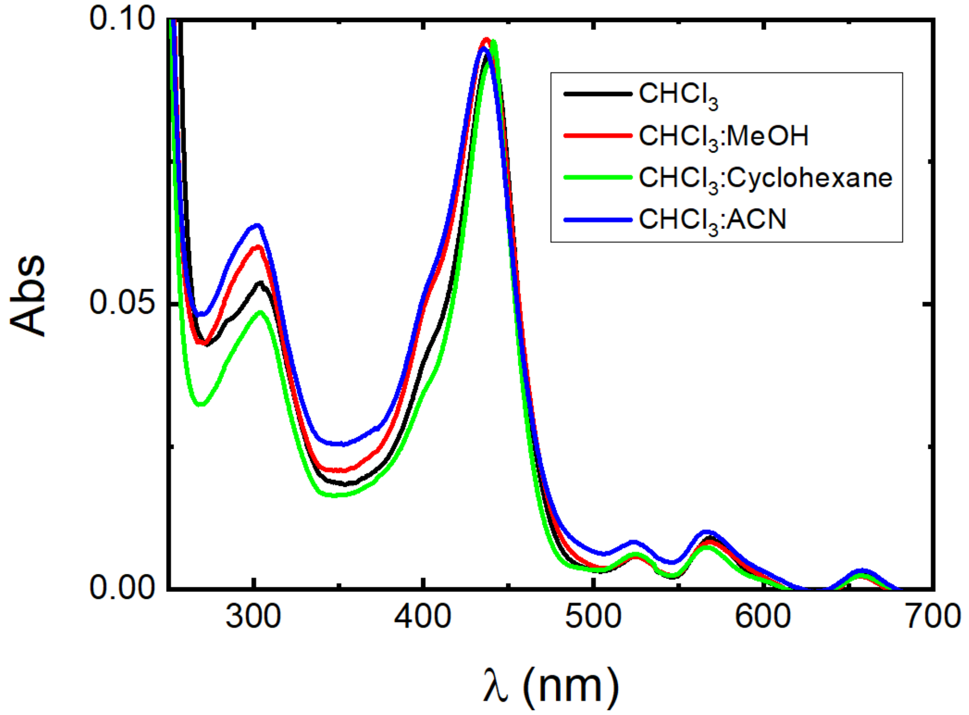


**Figure S5.** Absorption spectra of $H_2T(TPA)_4P$ ($5.2\times10^{-7}$ M) in $CHCl_3$ and in 1:1 (v:v) $CHCl_3$:CyHx, $CHCl_3$:MeOH and $CHCl_3$:$CH_3CN$ mixtures.

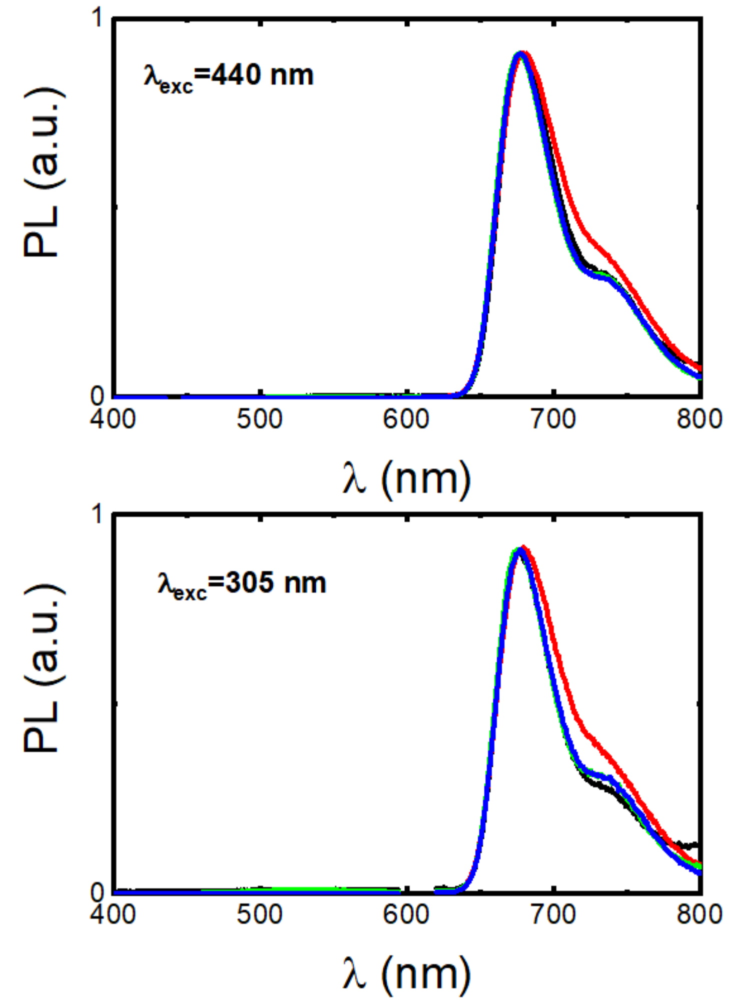


**Figure S6.** Normalized fluorescence emission spectra obtained at $\lambda_{exc}$= 440 nm (top) and 300 nm (bottom) of $H_2T(TPA)_4P$ ($5.2\times10^{-7}$ M) in $CHCl_3$ (—), $CHCl_3$:CyHx (—), $CHCl_3$:MeOH (—) and $CHCl_3:CH_3CN$ (—) solutions.

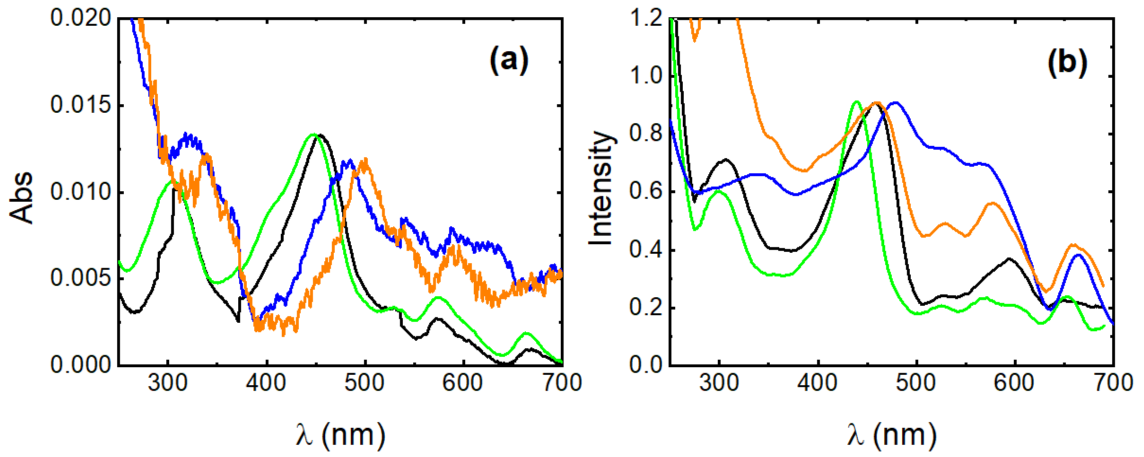


**Figure S7.** Normalized **(a)** Absorption and **(b)** excitation ($\lambda_{em}$= 680 nm) spectra of $H_2T(TPA)_4P$ films prepared by drop-cast of $1\times10^{-4}$ M solution in $CHCl_3$, $CHCl_3$:CyHx, $CHCl_3$:MeOH and $CHCl_3:CH_3CN$ solutions: Film 1 (—), Film 2 (—), Film 3 (—) and Film 4 (—), respectively.

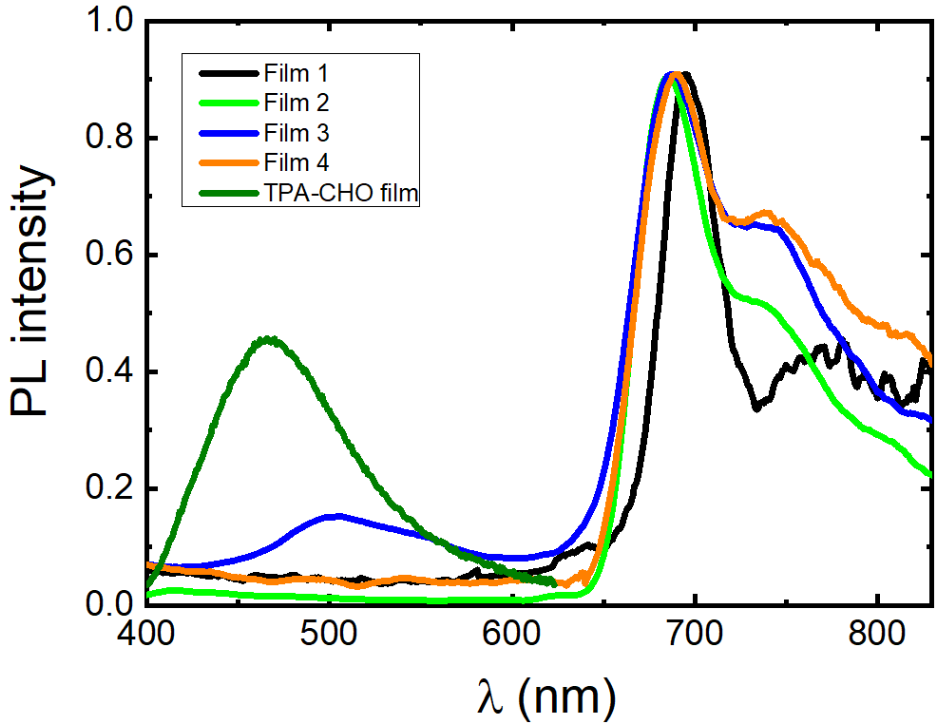


**Figure S8.** Normalized fluorescence emission spectra obtained at $\lambda_{exc}$= 300 nm of $H_2T(TPA)_4P$ thin films prepared by drop-cast of $1{\times}10^{-4}$ M solution in $CHCl_3$, $CHCl_3$:CyHx, $CHCl_3$:MeOH and $CHCl_3$:$CH_3CN$ solutions, Films 1 to 4, respectively. The emission spectra of a 4-(diphenylamino)benzaldehyde (TPA-CHO) film prepared through the drop-cast of $1{\times}10^{-4}$ M $CHCl_3$ solution is also presented.

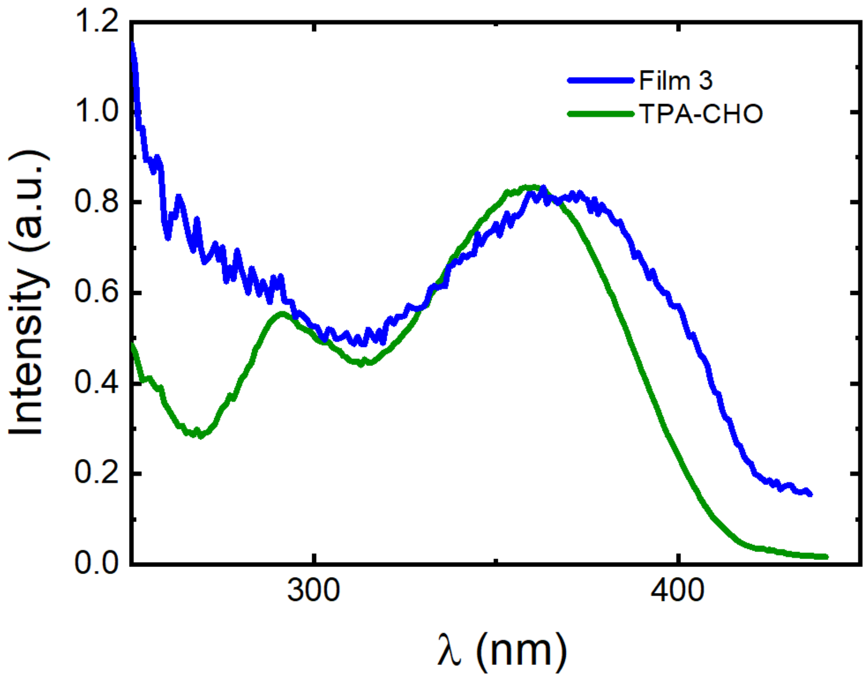


**Figure S9.** Normalized excitation spectra of **Film 3** and TPA-CHO ($1.0{\times}10^{-4}$ M) thin film, obtained with $\lambda_{exc}$= 450 nm.

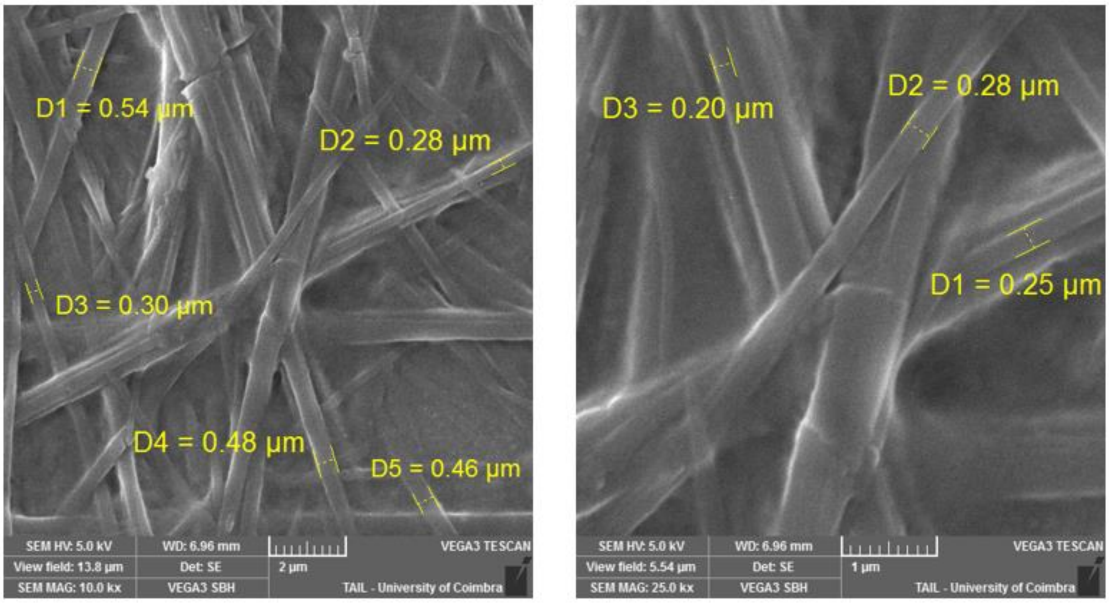


**Figure S10**. SEM of Film **1** prepared by drop cast of a solution of $H_2T(TPA)_4P$ in $CHCl_3$ ($1\times10^{-4}$ M).

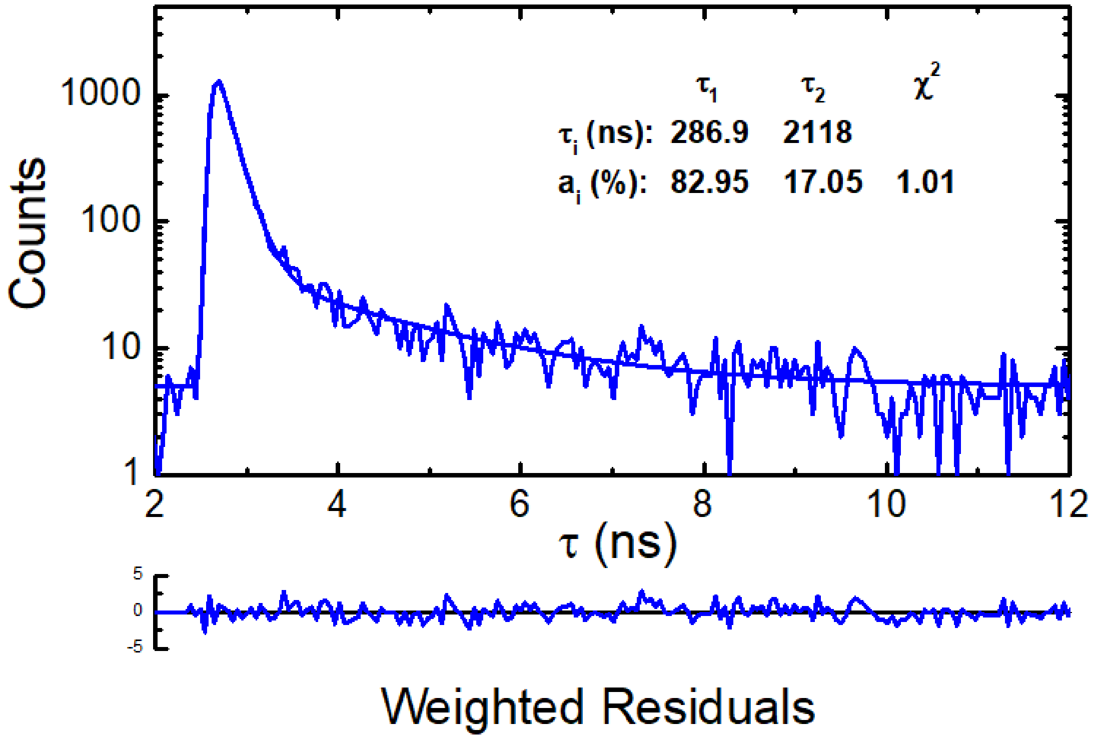


**Figure S11**. Single-pixel fluorescence decay of $H_2T(TPA)_4P$ aggregate retrieved from FLIM data. As an inset is shown the fluorescence decays times and pre-exponential factors obtained through the individual analysis of the decay using a bi-exponential function.

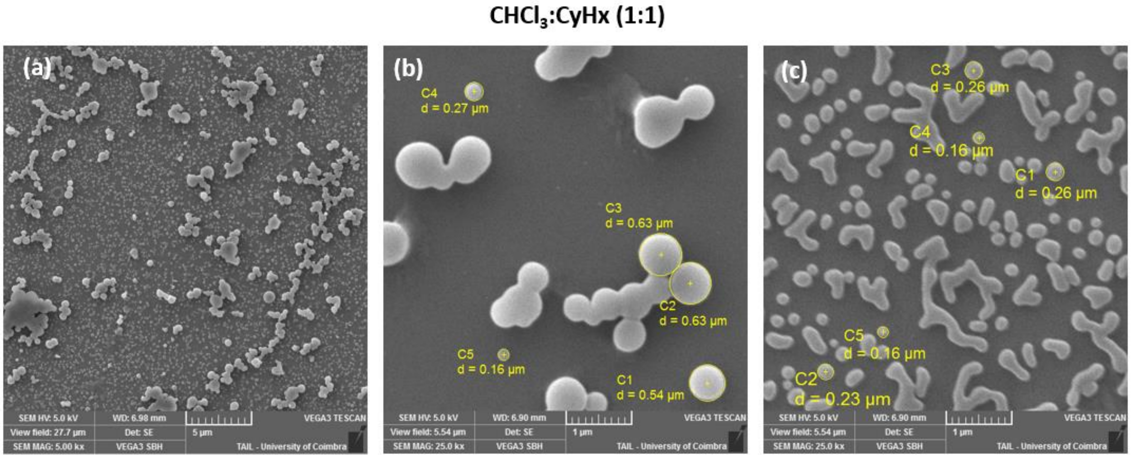


**Figure S12.** SEM of Film **2** prepared by drop cast of $H_2T(TPA)_4P$ in $CHCl_3$: CyHx (1:1 v:v) solution ($1{\times}10^{-4}$ M).

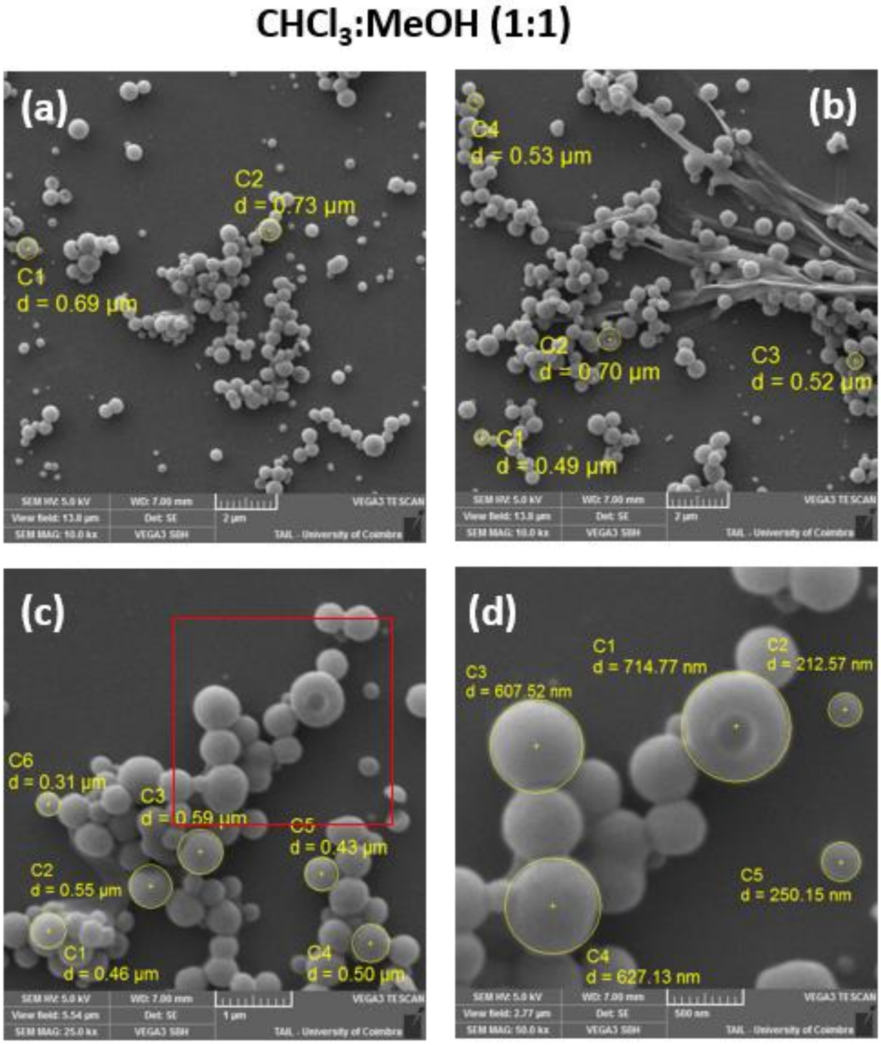


**Figure S13**. SEM of Film **3** prepared by drop cast of $H_2T(TPA)_4P$ in $CHCl_3$:MeOH (1:1 v/v) solution ($1{\times}10^{-4}$ M).

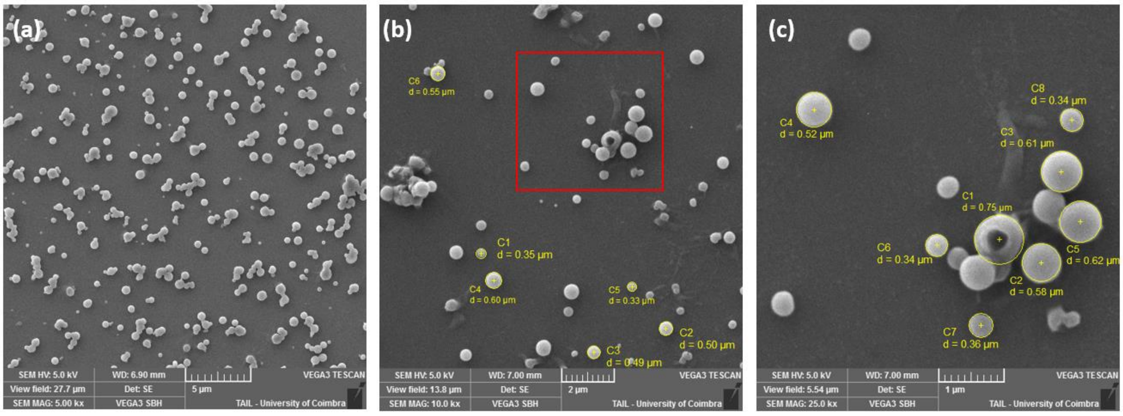


**Figure S14.** SEM of Film **4** prepared by drop cast of $H_2T(TPA)_4P$ in $CHCl_3$: $CH_3CN$ (1:1 v: v) solution ($1\times10^{-4}$ M).

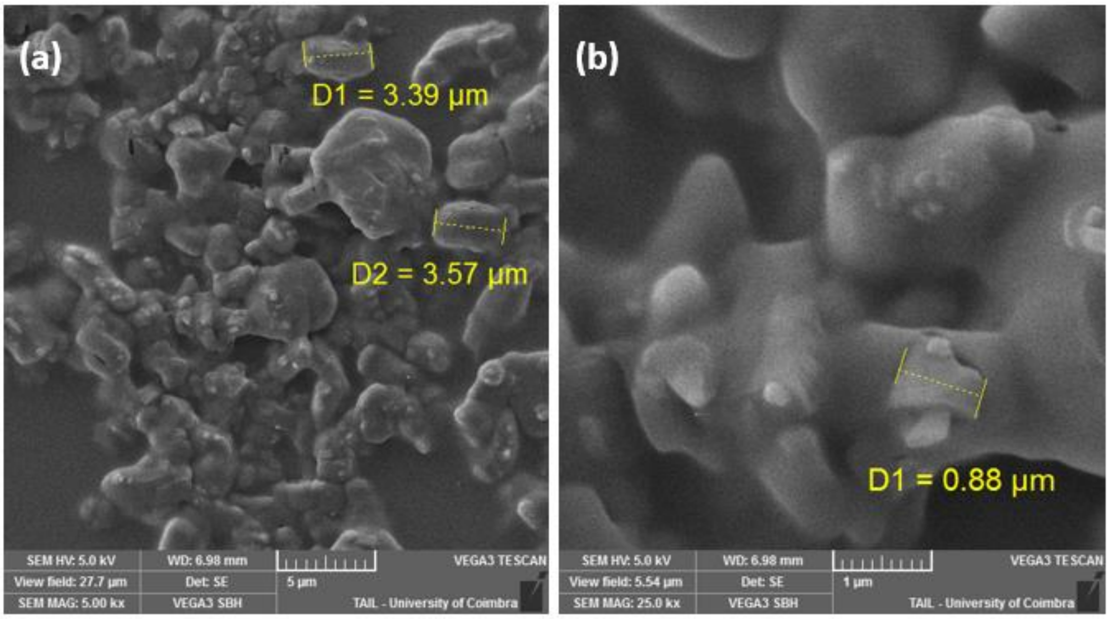


**Figure S15.** SEM of Film **5** prepared by spin-coating of a $H_2T(TPA)_4P$ solution ($1\times10^{-4}$ M) dissolved in Zeonex.

**$H_2T(TPA)_4P$**

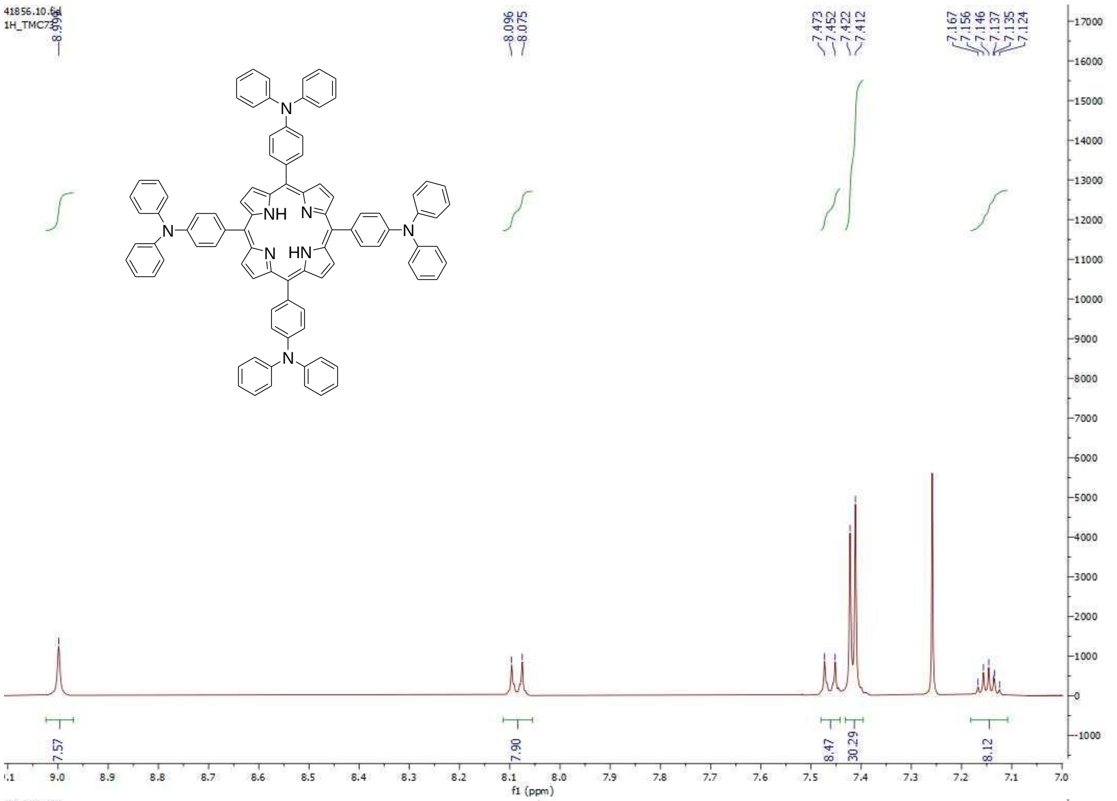

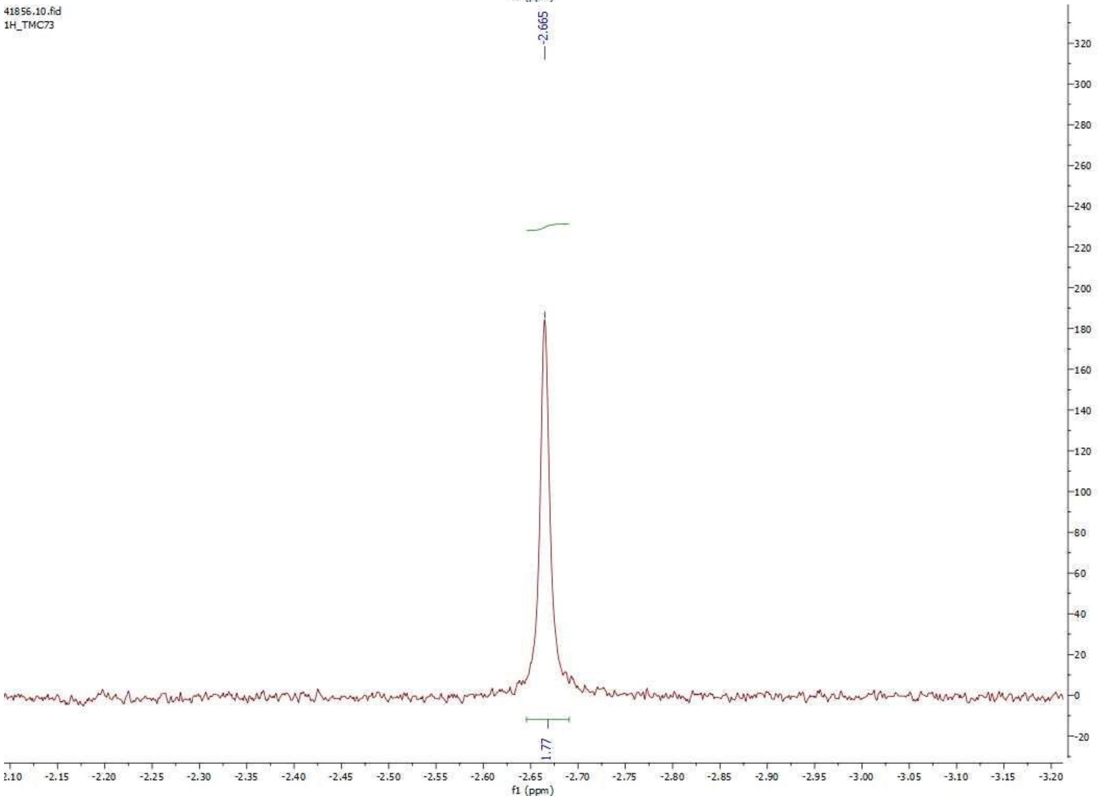

**Figure S16.** $^1$H-NMR spectrum of **$H_2T(TPA)_4P$** in $CDCl_3$.

REFERENCES

(1) Valeur, B., *Molecular Fluorescence: Principles and Applications*, 1st ed.; Wiley-VCH Verlag GmbH: New York, 2001.
(2) Sudha, K.; Sundharamurthi, S.; Karthikaikumar, S.; Abinaya, K.; Kalimuthu, P., Switching of Förster to Dexter Mechanism of Short-Range Energy Transfer in meso-Anthrylporphyrin. *J. Phys. Chem. C* **2017**, *121* (11), 5941-5948. DOI: 10.1021/acs.jpcc.6b13042
(3) Ventura, B.; Barigelletti, F.; Lodato, F.; Officer, D. L.; Flamigni, L., Energy transfer processes in electronically coupled porphyrin hetero-dyads connected at the β position. Phys. Chem. Chem. Phys. **2009**, *11* (13), 2166-2176. DOI: 10.1039/B819138G
(4) Montalti, M.; Credi, A.; Prodi, L.; Gandolfi, M. T., *Handbook of Photochemistry*, 3rd ed.; CRC Press: Boca Raton, 2006.
